# Optimizing potassium polysulfides for high performance potassium-sulfur batteries

**DOI:** 10.1038/s41467-024-45405-w

**Published:** 2024-02-02

**Authors:** Wanqing Song, Xinyi Yang, Tao Zhang, Zechuan Huang, Haozhi Wang, Jie Sun, Yunhua Xu, Jia Ding, Wenbin Hu

**Affiliations:** 1 School of Materials Science and Engineering, Tianjin Key Laboratory of Composite and Functional Materials Key Laboratory of Advanced Ceramics and Machining Technology (Ministry of Education), Tianjin University, Tianjin, China; 2 School of Materials Science and Engineering, Hainan University, Haikou, China; 3 School of Chemical Engineering and Technology, Tianjin University, Tianjin, China; 4 Joint School of National University of Singapore and Tianjin University, International Campus of Tianjin University, Binhai New City, Fuzhou, China

**Keywords:** Batteries, Batteries, Batteries, Materials chemistry

## Abstract

Potassium-sulfur batteries attract tremendous attention as high-energy and low-cost energy storage system, but achieving high utilization and long-term cycling of sulfur remains challenging. Here we show a strategy of optimizing potassium polysulfides for building high-performance potassium-sulfur batteries. We design the composite of tungsten single atom and tungsten carbide possessing potassium polysulfide migration/conversion bi-functionality by theoretical screening. We create two ligand environments for tungsten in the metal-organic framework, which respectively transmute into tungsten single atom and tungsten carbide nanocrystals during pyrolysis. Tungsten carbide provide catalytic sites for potassium polysulfides conversion, while tungsten single atoms facilitate sulfides migration thereby significantly alleviating the insulating sulfides accumulation and the associated catalytic poisoning. Resultantly, highly efficient potassium-sulfur electrochemistry is achieved under high-rate and long-cycling conditions. The batteries deliver 89.8% sulfur utilization (1504 mAh g^−1^), superior rate capability (1059 mAh g^−1^ at 1675 mA g^−1^) and long lifespan of 200 cycles at 25 °C. These advances enlighten direction for future KSBs development.

## Abstract (web summary)

Potassium-sulfur battery are promising materials for next-generation high energy, low cost batteries. Here the authors explore a tungsten based catalytic composite for optimizing potassium polysulfides and improve K-S electrochemistry in batteries

## Introduction

The development of efficient energy storage systems (ESS) is one key initiative for pursuing carbon neutrality worldwide^1^. Based on the high capacity of sulfur redox^2,3^ and the low potential of potassium metal^4^, potassium sulfur batteries (KSBs) have desirable theoretical energy density of 1023 Wh kg^−1^ thereby holding great promise for next-generation ESS. KSBs are intrinsically low in cost due to the abundance of sulfur and potassium. Nonetheless, some challenges around sulfur cathode significantly plague the KSB operation, such as the low sulfur utilization, the sluggish kinetics of potassium polysulfides (KPSs) conversion, the severe shuttling, and the difficult decomposition of solid end-products (K_2_S_*x*_, 1 ≤ *x* ≤ 3)^5^. These intractable impediments limit the capacity and lifespan of KSBs. Therefore, it is of vital importance to develop functional hosts accommodating the sulfur for addressing these issues.

Some pioneering efforts innovated functional sulfur hosts for KSBs. Most of the hosts are carbon-based, taking advantage of the good electrical conductivity, diverse surface chemistry, and versatile porosity of carbon^6,7^. The sulfur confinement is the primary function of the host. Physically confining sulfur in the carbon porosity is a popular strategy^8,9^. For instance, microporous carbons confining small molecular (S_1–3_) exhibited promising capacity and cyclability in KSBs. Nonetheless, the limitation of pore volume normally sacrificed the sulfur loading^7,10^. The carbon hosts employed were normally decorated by nitrogen moieties, which are effective of enhancing the sulfur anchoring via covalent bonding at heterointerfaces^11^. In addition, the chemical confinement strategy was also widely reported^12–14^. Sulfurized polyacrylonitrile (SPAN) utilizing the covalent sulfur-carbon bonds greatly inhibited KPSs shuttling^14–17^. The chemical confinement also includes the chemical adsorption between KPSs and certain polar species of the host, such as heteroatom groups, nanostructured compounds, atomic metal assemblies^7,18,19^. For instance, single metal atoms^18,20,21^ and metal carbides^22–25^ were reported providing adsorption effect towards polysulfides.

The function of catalytic conversion towards KPSs in sulfur host attracted increasing attention in recent years. Some highly enlightening studies introduced catalytic sites in the hosts to improve the KPSs conversion kinetics, especially the solid-state reactions including K_2_S oxidation^26^. Metallic single atoms^18,26^ and metallic atom clusters^27^ were reported as electrocatalytic species, which suppressed the long-chain KPSs shuttling and reduced the polarization of solid-state sulfide conversion, thereby enhancing the sulfur utilization, rate and cycling capability. The design of catalytic materials for KPSs can be inspired by the prior achievements in Li/Na-sulfur batteries. The metal element in various forms (e.g. single atom, atomic cluster, compounds) that can deliver general catalytic capability towards both lithium and sodium polysulfides deserves particular attention. For example, tungsten is catalytically active in Li-S batteries (tungsten single atom^20^, tungsten carbide^23–25^, tungsten sulfide^28,29^) and Na-S batteries (tungsten nanoparticle^30^). The successful applications of tungsten-based catalysts in metal sulfur batteries were largely ascribed to the special electronic structure of tungsten atomic assemblies and tungsten compounds^31^. These insights are highly instructive for KSBs explorations.

The previous researches reveal the importance of introducing catalytically active sites in sulfur hosts for KPSs conversion. Considering the complex potassium-sulfur electrochemistry, more factors should be considered beyond the catalytic conversion. First, sulfur is essentially confined in the carbon porosity, which distributes ubiquitously in the host. On the contrary, the isolate nanostructured catalytic species can only locate disjunctively^19,32^. Therefore, there is an inherent mismatch in the distribution of sulfur and catalytic sites, which makes the KPSs migration among the catalytic sites highly necessary. More importantly, the reduction products of solid-state KPSs conversion are highly insulating and difficult to decompose^33^, which can easily accumulate over the catalytic sites and cause catalytic poisoning^34,35^. Therefore, the migration of K_2_S/K_2_S_2_ away from the catalytic sites is important for preventing the expansion of electronically inactive areas in the cathode, especially under high current density and aggressively repeated charge/discharge conditions. The above considerations suggest that KPSs migration can play an important role in KSBs, which unfortunately has been totally overlooked. This motivated us to develop sulfur host with combined functionalities of accelerating KPSs migration and catalyzing KPSs conversion for pursuing high-performance KSBs.

In this study, to implement the principle of optimizing KPSs behavior, the coupling of tungsten single atom (W_SA_) and tungsten carbide (W_2_C) is designed based on density functional theory (DFT) calculation. Creating different coordination environments for tungsten in metal organic framework precursor drives the symbiosis of W_SA_ and W_2_C on nitrogen-doped carbon (NC) upon pyrolysis. Employing the obtained W_SA_-W_2_C@NC as sulfur host enables the KSBs to deliver ultra-high capacity of 1504 mAh g^−1^, rate performance (1059 mAh g^−1^ at 1675 mA g^−1^) and stability (cycling over 200 cycles). Combined spectroscopic characterizations and theoretical computations revealed the critical role of KPSs optimization in avoiding inert sulfides accumulation and catalytic poisoning, as well as enhancing rate and cycling performances of KSBs.

## Results

### Theoretical guidance and screening for sulfur host design

**Fig. 1:**
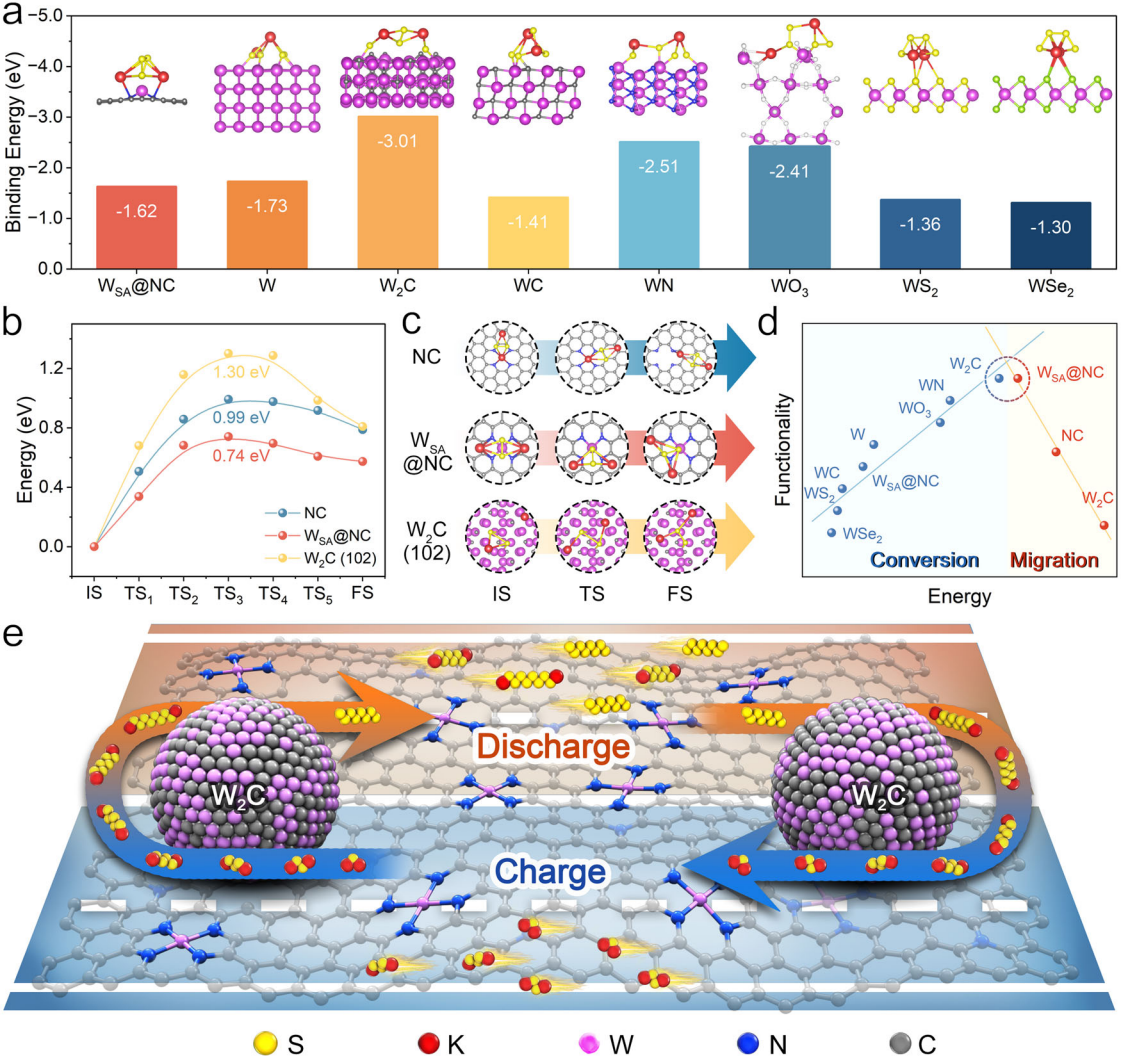
Theoretical guidance and screening for sulfur host design. **a** The binding energies of K_2_S_4_ on W_SA_@NC, W metal (110), W_2_C (102), WC (101), WN (112), WO_3_ (100), WS_2_ (002) and WSe_2_ (006) and the corresponding configurations (insets). **b** The energy profiles of K_2_S_2_ migration on NC, W_SA_@NC, and W_2_C (102). **c** The calculated initial, transition and final states of K_2_S_2_ migration. **d** Volcano plot of KPS conversion and migration functionalities with respect to specific energy values. **e** Schematic demonstrating the design principle of the W_SA_-W_2_C composite based sulfur host for efficient sulfur redox in KSBs guided by the theoretical calculation. Source data are provided as a Source Data file.

The interactions between the functional species in sulfur hosts and potassium polysulfides (KPSs) are comprehensively investigated to acquire the principles of the host design^21,36^. Regarding to the catalytic conversion functionality towards KPSs, the chemical binding of key polysulfide on the sulfur host is the primary factor^21,36,37^. For saving trial-and-error experiments, density functional theory (DFT) calculation was first conducted to screen the optimal catalytic species. The stoichiometric middle point of the whole KPSs conversion, i.e. K_2_S_4_, was employed as the representative for the binding energy calculations. Figure 1a lists the tungsten species including tungsten metal/carbides/nitride/oxide/sulfide/selenide and nitrogen coordinated single atom tungsten (W_SA_) for the theoretical screening. The specific crystal planes of tungsten species to interact with K_2_S_4_ were determined based on the maximum surfaces of the Wulff configurations (Supplementary Fig. 1 and Supplementary Table 1). The optimized binding configurations of K_2_S_4_ on tungsten species and corresponding binding energy values were displayed in Fig. 1a. W_2_C (102) was screened out by the highest binding energy for K_2_S_4_ (−3.01 eV).

Apart from the KPSs conversion catalysis, the facile KPSs migration over sulfur hosts should be considered with equal importance due to the mismatch between ubiquitously distributed sulfur and disjunctively distributed catalytic sites. Among aforementioned tungsten species, only W_SA_ can achieve the atomically homogeneous dispersion on the host, because the compounds, even being nanostructured, can only exist as spatially discrete islands. Therefore, we calculated the migration kinetics of K_2_S_2_ (key intermediate of solid-state KPSs conversion and midpoint of S^0^/S^2-^ redox) on W_2_C, W_SA_ modified nitrogen-doped carbon (W_SA_@NC) and NC. Figure 1b demonstrates the initial, transition and final states of K_2_S_2_ migration on NC, W_SA_@NC, and W_2_C (102). The corresponding energy barriers for K_2_S_2_ migrations are determined to be 0.99, 0.74, and 1.30 eV. Evidently, W_SA_ could effectively lower the energy barrier of K_2_S_2_ migration, enabling facile transfer of solid-state KPSs^36,38^.

The comprehensive consideration of the catalytic KPSs conversion and facilitated KPSs migration results in the volcano-type relationship in Fig. 1d. At the vertex of the volcano, W_2_C and W_SA_ should be selected as dual functional components in sulfur host. Figure 1e is the scheme demonstrating the design principle of the sulfur host guided by the theoretical calculation. W_2_C nanocrystals provide isolated catalytic sites for KPSs conversion, while W_SA_ modified carbon substrate act as highways for fast KPSs transportation. During the discharge/charge processes, KPSs could be fed to W_2_C sites for catalytic conversion, and afterwards the reaction products could also be promptly released away from W_2_C sites. This mechanism can effectively address the distribution mismatch of sulfur and catalytic sites. Moreover, the facile solid-state KPSs migration surrounding W_2_C is expected to be highly conducive to the durability of the catalytic sites by avoiding catalytic poisoning.

### Fabrication and characterization of sulfur hosts and sulfur cathodes

**Fig. 2:**
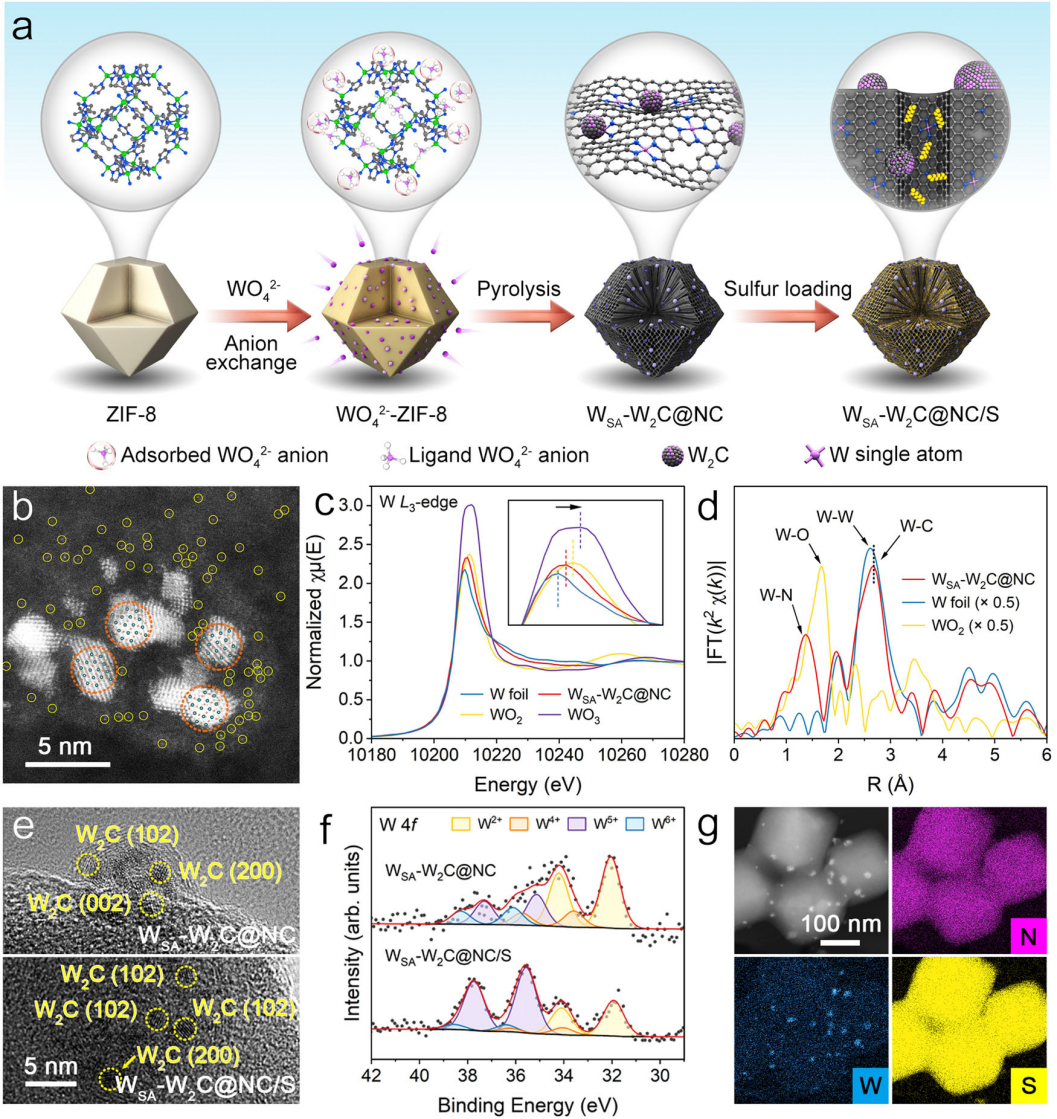
Synthesis and characterization of W_SA_-W_2_C@NC and W_SA_-W_2_C@NC/S. **a** Schematic synthesis process of W_SA_-W_2_C@NC and W_SA_-W_2_C@NC/S. **b** HAADF-STEM image of W_SA_-W_2_C@NC. **c**–**d** XANES (**c**) and EXAFS (**d**) spectra of W *L*_3_-edge for W_SA_-W_2_C@NC and W foil, WO_2_, WO_3_ references. **e** HRTEM images of W_SA_-W_2_C@NC and W_SA_-W_2_C@NC/S. **f** High-resolution W 4 *f* XPS spectra for W_SA_-W_2_C@NC and W_SA_-W_2_C@NC/S. **g** HAADF-STEM image and corresponding EDS mapping of W_SA_-W_2_C@NC/S. Source data are provided as a Source Data file.

To experimentally implement the conceived sulfur host based on theoretical guidance, we employed a sophisticated method to construct composite of tungsten single atoms and tungsten carbide nanocrystals on nitrogen-doped porous carbon (termed W_SA_-W_2_C@NC). Figure 2a demonstrates the synthesis procedure. ZIF-8 nanoparticles were prepared as nitrogen containing carbon precursor for loading tungsten. WO_4_^2-^ anions as tungsten sources were introduced into ZIF-8 matrix through solvothermal treatment. In the obtained WO_4_^2-^-ZIF product, a portion of WO_4_^2-^ anions incorporate into the organic ligands of zinc atoms in ZIF-8 through an anion exchange process. Meanwhile, another portion of WO_4_^2-^ anions adsorbed on the ZIF-8 surface by the electrostatic effect. Therefore, two different coordination environments were created for tungsten in WO_4_^2-^-ZIF precursor, which induced different tungsten coalescence processes during the subsequent pyrolysis. The WO_4_^2-^ anions coupling with the organic ligands of ZIF-8 underwent a spatial confinement pyrolysis, which transmute into nitrogen coordinated single tungsten atoms (W_SA_). WO_4_^2-^ anions on ZIF-8 surface agglomerated and formed tungsten carbides in the carbon rich atmosphere. ZIF-8 matrix was carbonized into nitrogen-doped porous carbon, supporting the W_SA_ and W_2_C species.

The obtained W_SA_-W_2_C@NC inherited the polyhedron morphology (Supplementary Fig. 2). Aberration-corrected high angle annular dark-field scanning transmission electron microscopy (HAADF-STEM) and high-resolution transmission electron microscopy (HRTEM) images (Fig. 2b, Supplementary Figs. 3 and 4a) demonstrated that tungsten species in W_SA_-W_2_C@NC include the W_2_C nanocrystals (dashed circles) and single tungsten atoms (solid circles). Single tungsten atoms distribute over the whole NC substrate and embrace W_2_C nanocrystals. Of note, the small-size and relatively low loading W_2_C nanocrystals cannot be detected by XRD (Supplementary Fig. 5). For determining the optimal content of W_SA_-W_2_C in the hosts, specimens with higher W_SA_-W_2_C content (W_SA_-W_2_C-H@NC) and lower W_SA_-W_2_C content (W_SA_-W_2_C-L@NC) were also prepared. The W_SA_ and W_2_C species in these two control specimens could be well identified by SEM, HRTEM and HAADF-STEM images (Supplementary Fig. 6).

W_2_C@NC was prepared by eliminating the solvothermal anion exchange process during synthesis, in which case only electrostatically adsorbed WO_4_^2-^ existed in the precursor. In HRTEM and HAADF-STEM images (Supplementary Figs. 4b and 7), only tungsten carbide (W_2_C) nanocrystals were present with scarcely any single tungsten atom observed. Similar to W_SA_-W_2_C@NC, the W_2_C nanocrystals are too small to be detected by XRD (Supplementary Fig. 5). This result verifies the importance of creating different coordination environments of tungsten in the precursor for achieving the symbiosis of W_SA_ and W_2_C upon pyrolysis. Baseline of nitrogen-doped porous carbon (NC) was prepared by directly carbonizing tungsten-free ZIF-8. HRTEM images and selected area electron diffraction (SAED) patterns confirmed the amorphous tissue of NC (Supplementary Fig. 8).

The electronic structures and atomic configurations of tungsten species in W_SA_-W_2_C@NC were verified by X-ray absorption near-edge structure (XANES) and Fourier transform extended X-ray absorption fine structure (EXAFS) spectra. In Fig. 2c, the white line peak in the W *L*_3_-edge XANES spectrum of W_SA_-W_2_C@NC lies between tungsten metal foil and WO_2_, indicating an average valence state between 0 and +4. EXAFS spectrum demonstrates the distinct W‒N peak at ~1.35 Å and W‒C peak at ~2.67 Å^39–41^, which verifies the coexistence of single tungsten atoms and tungsten carbides in W_SA_-W_2_C@NC.

Employing the aforesaid hosts, W_SA_-W_2_C@NC/S, W_2_C@NC/S, and NC/S were prepared by melting-impregnation method. The products retained the morphologies of the pristine hosts (Supplementary Fig. 9). No isolated sulfur particle could be observed, suggesting the full infiltration of sulfur in the hosts. According to HRTEM images, the size and crystal structures of the tungsten carbides remain unchanged after sulfur loading for both W_SA_-W_2_C@NC/S (Fig. 2e) and W_2_C@NC/S (Supplementary Fig. 10). Control specimens of W_SA_-W_2_C-H@NC/S and W_SA_-W_2_C-L@NC/S display the same phenomenon, as revealed in Supplementary Fig. 11. X-ray photoelectron spectroscopy (XPS) demonstrated the interaction between sulfur and the host^28,42,43^. In the W 4 *f* spectra (Fig. 2f), the sulfur loading induced an elevated proportion of W^5+^ and an increase in average valence from +3.43 to +4.07 for W_SA_-W_2_C@NC/S. By contrast, the W 4 *f* in W_2_C@NC/S hardly changed (Supplementary Fig. 12). This phenomenon indicates that the interaction between sulfur and atomic-level homogeneously dispersed W_SA_ is more pronounced, which also suggests the sulfur distribution all over the entire host.

The HAADF-STEM images and elemental mappings in Fig. 2g and Supplementary Fig. 13 revealed that sulfur uniformly distributed in W_SA_-W_2_C@NC, W_2_C@NC and NC hosts. SAED patterns (Supplementary Fig. 14) display no diffraction spots or rings of crystalline sulfur, suggesting the amorphous texture of sulfur in these specimens. The absence of cyclo-S_8_ characteristic peaks in the Raman spectra (Supplementary Fig. 15) agreed with the SAED data^22^. According to the nitrogen adsorption-desorption isotherms analyses (Supplementary Fig. 16), the significantly decreased surface areas of the composites as compared to pristine hosts (e.g. 15.96 m^2^ g^−1^ of W_SA_-W_2_C@NC/S versus 706.60 m^2^ g^−1^ of W_SA_-W_2_C@NC) confirmed the effective sulfur infusion. Moreover, the sulfur hosts all provide microporosities for sulfur accommodation, which should be the essential reason for the amorphous structure of the impregnated sulfur^44^.

### Electrochemical performances and sulfur redox mechanisms

**Fig. 3:**
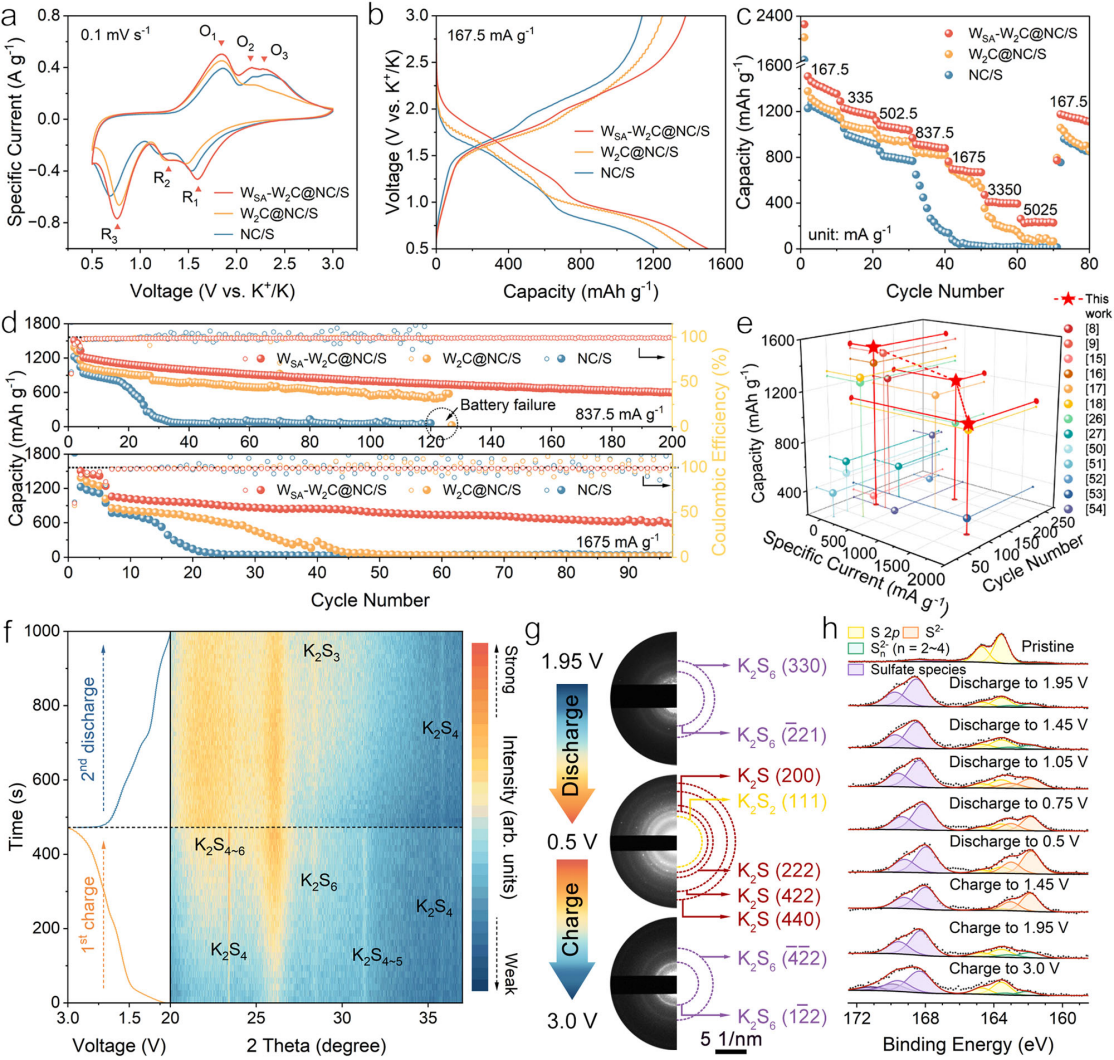
Electrochemical performances and sulfur redox mechanism. **a** CV curves of KSBs employing W_SA_-W_2_C@NC/S, W_2_C@NC/S, and NC/S cathodes. **b**–**d** GCD profiles curves at 167.5 mA g^−1^ (**b**) rate-performance (**c**) and cyclability (**d**) of KSBs employing three cathodes. **e** Comparison of specific capacity, rate and cycling performance of W_SA_-W_2_C@NC/S cathode with the state-of-the-art cathodes for KSBs reported. **f** In-situ XRD of W_SA_-W_2_C@NC/S cathode in the 1^st^ charge and 2^nd^ discharge processes. **g** Ex-situ SAED patterns of W_SA_-W_2_C@NC/S cathode at various discharge/charge states. **h** Ex-situ S 2*p* XPS spectra of W_SA_-W_2_C@NC/S cathode at selected discharge/charge states. Source data are provided as a Source Data file.

W_SA_-W_2_C@NC/S utilizing the host containing functional species of W_SA_ and W_2_C is expected to be high-performance cathodes for KSBs. First, W_SA_-W_2_C@NC and W_2_C@NC demonstrated much stronger adsorption capability towards KPSs than that of tungsten-free NC, as proved by the ultraviolet-visible (UV-vis) spectra of K_2_S_6_ solutions with different host materials (Supplementary Fig. 17)^45,46^. This phenomenon agrees well with the calculation demonstrated in Fig. 1. After 12 h, all K_2_S_6_ species in the solution were thoroughly adsorbed by W_SA_-W_2_C@NC and W_2_C@NC hosts, as evidenced by the corresponding spectra overlapping with pure DME solvent. To understand the sulfur redox in W_SA_-W_2_C@NC/S, W_2_C@NC/S and NC/S cathodes, cyclic voltammetry (CV) measurements were conducted. As shown in Supplementary Fig. 18, in the first cathodic scan of W_SA_-W_2_C@NC/S, there is a strong peak at 0.836 V combining the SEI formation^9,19,22,32^ and sulfur activation processes^8,47–49^. The sulfur activation describes the potassiation of pristine sulfur, which probably needs to overcome higher energy barrier than the following potassiation processes^22,44,49^. Therefore, the cathodic peak of the sulfur potassiation in the 1^st^ cycle CV appeared at lower onset voltage than that in the following cycles^9,19,22^. Reduction peaks at 1.594 (R_1_), 1.329 (R_2_), and 0.761 V (R_3_) appeared in the 2^nd^ cycle (Fig. 3a). In the anodic scan, oxidation peaks present at 1.844 (O_1_), 2.159 (O_2_), and 2.272 V (O_3_). W_2_C@NC/S and NC/S exhibit similar CV shapes as W_SA_-W_2_C@NC/S, but deliver lower response specific currents and larger polarizations. This phenomenon suggests the faster sulfur redox kinetics for W_SA_-W_2_C@NC/S than the other two cathodes.

The galvanostatic charge/discharge (GCD) curves of the three cathodes (2^nd^ cycle) were displayed in Fig. 3b. The W_SA_-W_2_C@NC/S cathode delivered a high discharge capacity of 1504 mAh g^−1^ based on the thermogravimetric analysis (TGA) determined sulfur content (40.56%) (Supplementary Fig. 19). This reversible capacity corresponds to 89.8% sulfur utilization, which is the highest value reported by far. The counterpart values for W_2_C@NC/S and NC/S are 82.8% and 73.3%, respectively, highlighting the advantage of W_SA_-W_2_C composite in enhancing the electrochemical activity of sulfur. The discrepancy in sulfur content among three cathodes is below 3.4%, which is at the lowest level in the field (Supplementary Table 2). The cathodes with such sulfur content discrepancy deliver negligible difference in electrochemistry (Supplementary Fig. 20). The absolute sulfur contents above 40% are higher than the reported sulfur-microporous carbon composites and on par with SPANs (Supplementary Table 3). The extremely low capacities of the pure hosts (Supplementary Fig. 21) verified that the capacities of cathodes are provided by the reversible sulfur redox. Of note, the three specimens exhibited highly similar nitrogen moieties in terms of nitrogen types and relative ratios (Supplementary Fig. 22 and Supplementary Table 4). Therefore, the different electrochemical performances of three cathodes should be essentially stemmed from the tungsten species. W_SA_-W_2_C@NC/S electrodes with high sulfur loading of 2.86 and 3.61 mg cm^–^^2^ delivered slightly lower capacities of 1477.8 and 1221.9 mAh g^–^^1^ (Supplementary Fig. 23), which are higher than most previously reported cathodes with low sulfur loading (Supplementary Table 3). Control cathodes of W_SA_-W_2_C-H@NC/S and W_SA_-W_2_C-L@NC/S delivered capacities of 1274.4 and 1416.1 mAh g^−1^ (Supplementary Fig. 24), suggesting W_SA_-W_2_C@NC/S has the optimal W_SA_-W_2_C content.

Regarding to the rate capability (Fig. 3c), W_SA_-W_2_C@NC/S cathode exhibited capacities of 914, 697, 408, and 223 mAh g^–^^1^ at current densities of 838, 1675, 3350, 5025 mA g^–^^1^, respectively. W_2_C@NC/S cathodes delivered lower capacities than W_SA_-W_2_C@NC/S at all rates. It is worth special noting that the capacity fast decayed for W_2_C@NC/S at 3350–5025 mA g^–^^1^, resulting in cell failure at high rate. This issue was more severe for NC/S cathode, as indicated by the rapid cell failure at 837.5 mA g^–^^1^. Considering the different constitutions of tungsten species for W_SA_-W_2_C@NC and W_2_C@NC, it is reasonable to speculate that the cooperation between W_SA_ and W_2_C plays a critical role in maintaining the cell operation because more facile sulfur redox kinetics are required at high specific currents.

The cycling performance were also measured in addition to the rate tests. As shown in Fig. 3d, after 5 activation cycles at 167.5 mA g^–^^1^, W_SA_-W_2_C@NC/S delivered capacities of 1214 and 1059 mAh g^–^^1^ at 837.5 and 1675 mA g^–^^1^, respectively. Afterwards, the cathodes kept stable cycling for 200 and 97 cycles, resulting in capacity retention ratio of 49.4 and 56.8%. W_SA_-W_2_C-H@NC/S and W_SA_-W_2_C-L@NC/S showed inferior cyclability, again proving the optimal content of W_SA_-W_2_C in W_SA_-W_2_C@NC (Supplementary Fig. 24). For comparison, W_2_C@NC/S cathode can last for only 127 and 40 cycles at 837.5 and 1675 mA g^–^^1^. The cell failure occurred even earlier for NC/S cathode, i.e. at 32^nd^ and 20^th^ cycles. The cell failure can only be postponed by cycling the cathodes at very slow rate (Supplementary Fig. 25). The changes in resistances in the cells provide another aspect reflecting the diversity in cyclability of the cathodes (details in Supplementary Fig. 26 and Table 5). Combining the rate and cycling performance, it could be concluded that certain injurious process continuously deteriorated sulfur redox property and finally damaged the cell, which can be aggravated at higher specific currents. More importantly, the synergy of the functionalities of W_SA_ and W_2_C can significantly alleviate this negative factor.

It is instructive to compare W_SA_-W_2_C@NC/S based KSB with the state-of-the-art KSBs reported. As shown in Fig. 3e and Supplementary Table 3, KSBs employing SPAN cathodes^15–17^, small-molecular sulfur cathodes^8,9,50^, polysulfide catholyte^51^ and cyclo-S_8_ cathodes^18,26,27,52–54^ are all included for comprehensive comparison. Remarkably, W_SA_-W_2_C@NC/S cathode reaches the highest sulfur utilization and best rate performance. It is worth special noting that the cyclability of W_SA_-W_2_C@NC/S cathode is comparable to that of most robust SPAN cathodes, suggesting the advantages of W_SA_-W_2_C@NC/S for practical KSBs.

To pursue insights of the K-S redox mechanism, in-situ XRD, ex-situ SAED and XPS were conducted to investigate the W_SA_-W_2_C@NC/S cathode during charge and discharge. Per Fig. 3f, as the charging process proceeded, the peaks of K_2_S_6_ emerged and gradually strengthened at the expense of K_2_S_4_ peak. In the following discharge process, the decrease of K_2_S_4–6_ peak intensity and increase of K_2_S_3_ peak intensity can be observed, suggesting the reversible conversion between K_2_S_6_ and K_2_S_3_ in W_SA_-W_2_C@NC/S cathode. The diffraction signals of K_2_S_2_ and K_2_S cannot be easily distinguished in the in-situ XRD patterns probably due to the short time for collecting each XRD pattern and the small size of target phases. Thus, SAED was used to further identify the reaction products at various discharge/charge states (Supplementary Fig. 27). Starting from the amorphous halo for pristine cathode (Supplementary Fig. 28), a bright diffraction ring corresponding to K_2_S_6_ appeared upon discharge (Fig. 3g). As the discharge continued, K_2_S_6_ ring gradually diminished (Dis-1.45 V). Diffraction rings ascribed to K_2_S phase appeared and became stronger (Dis-1.05/0.75 V). At the lowest discharge voltage of 0.5 V, only strong diffraction rings of K_2_S and relatively weak diffraction ring of K_2_S_2_ existed, suggesting the K_2_S and minor K_2_S_2_ as the discharge end products. During the reversible charge process, diffraction rings ascribed to K_2_S_4_ first appeared (Cha-1.45 V). Upon further charging, K_2_S_6_ reappeared (Cha-1.95/3.0 V), suggesting the fully reversible conversion from K_2_S to K_2_S_6_ in W_SA_-W_2_C@NC/S cathode.

Ex-situ XPS characterizations revealing valence change of sulfur were also conducted as supplement analyses on KPSs conversion. In Fig. 3h, a clear increase of S_n_^2-^ (163.4 eV, 162.2 eV) and S^2-^ (163.0 eV, 161.8 eV) components at the expense of S^0^ (164.7 eV, 163.5 eV) can be observed in discharge^55,56^. S^2-^ started to convert to S_n_^2-^ as charge back to 1.95 V. At the cut-off charge voltage of 3.0 V, both S_n_^2-^ and S^2-^ completely converted to S^0^. The XPS observations are highly consistent with the analyses derived from in-situ XRD and ex-situ SAED patterns.

The sulfur redox process can be resolved based on the systematic spectroscopic data. With regard to the CV curve in Fig. 3a, R_1_/R_2_ should be ascribed to the successive conversion of long-chain to short-chain KPSs, and R_3_ is associated with the reduction of short-chain KPSs into K_2_S. In the anodic scan, O_3_/O_2_, and O_1_ should be ascribed to the long-chain KPSs oxidation and solid-state KPSs oxidation, respectively. Comparing the peak intensities in the CV curves, the prominent long-chain KPSs conversion for NC/S, and facile short-chain KPSs conversion for W_2_C@NC/S can be well distinguished. Meanwhile, W_SA_-W_2_C@NC/S cathode exhibited the most facile kinetics in all the steps of long-/short-chain KPSs conversion.

### Sulfur redox kinetics under synergy of KPS conversion and migration

**Fig. 4:**
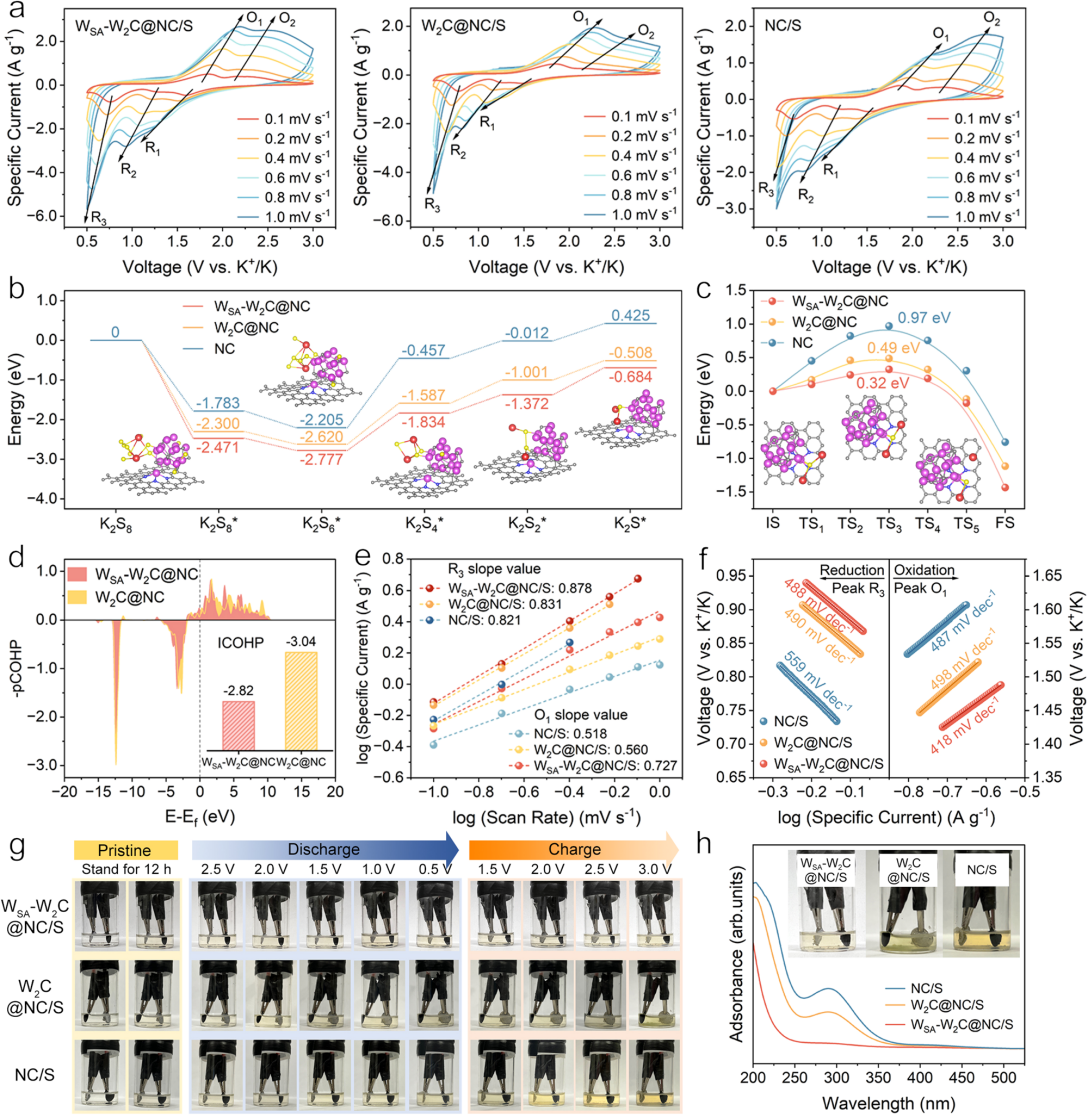
Sulfur redox kinetics under synergy strategy. **a** Multi-rate scan CV curves of KSBs employing W_SA_-W_2_C@NC/S, W_2_C@NC/S, and NC/S cathodes. **b** Gibbs free energy profiles of sulfur reduction processes on NC, W_2_C@NC, and W_SA_-W_2_C@NC hosts. **c** Energy profiles for K_2_S dissociation on W_SA_-W_2_C@NC, W_2_C@NC and NC. **d** Projected crystal orbital Hamilton population (pCOHP) diagrams of W‒S bonds linking K_2_S and W_SA_-W_2_C@NC, W_2_C@NC (insert shows ICOHP values). **e** Linear relationship of log (specific current) vs. log (scan rate) of peaks R_3_ and O_1_ for these three cathodes. **f** Tafel slope of peaks R_3_ and O_1_ derived from CV curves at 0.2 mV s^−1^. **g** Visual cells under different states employing W_SA_-W_2_C@NC/S, W_2_C@NC/S, NC/S cathodes and potassium foil anodes. **h** UV-vis spectra and the optical photos (inset) of visual cells employing different cathodes at the end of charge process. Source data are provided as a Source Data file.

Based on the index of the redox peaks on CV, more in-depth kinetic analysis of the cathodes can be conducted according to multi-rate CV scanning. The different trends of response specific currents and voltage polarizations as a function of scan rate reflect the kinetics of specific sub-steps of sulfur redox (Fig. 4a). First, the comparison of R_1_/R_2_ specific currents and polarizations for W_2_C@NC/S and NC/S demonstrated that W_2_C catalytic site had no significant promotion on the long-chain KPSs conversion. W_SA_-W_2_C@NC/S cathode delivered ca. 25% higher R_1_ specific current, which may probably be attributed to the facilitated long-chain KPSs migration across the carbon matrix by W_SA_ moieties. The most facile long-chain KPSs reduction kinetics enabled by W_SA_-W_2_C can also be demonstrated by the CV profiles of K_2_S_6_ based symmetric cell (Supplementary Fig. 29)^57–59^.

Regarding to the R_3_ peak ascribed to the solid-state KPSs reduction, W_SA_-W_2_C@NC/S displayed the lowest polarization and highest response specific current, suggesting the most facile kinetics of solid-state K_2_S_4_-K_2_S conversion. W_2_C@NC/S displays lower R_3_ specific current than that of W_SA_-W_2_C@NC/S, revealing the synergy of W_SA_ and W_2_C in enhancing solid-state reaction kinetics. Comparing the characteristics of R_3_ for W_2_C@NC/S and NC/S, W_2_C@NC/S exhibited an accelerated R_3_ redox, as indicated by the larger response specific currents than that of NC/S. The R_3_ peak for NC/S is much weaker especially at high scan rate suggesting the difficulty of solid-state KPSs conversion to reach K_2_S_2_/K_2_S without the catalytic effect of W_2_C nanocrystals.

To reveal the origin of the different sulfur reduction kinetics for the three cathodes, the Gibbs free energy changes along the sulfur reduction pathways were calculated. Figure 4b demonstrates the thermodynamically stable configurations of various KPSs binding with different hosts and the corresponding free energy profiles. It can be distinguished that the reaction sub-steps from K_2_S_8_ to K_2_S_6_ are exothermic for three cathodes. The successive sub-steps of reduction (from K_2_S_6_ to K_2_S) are all endothermic. The maximum energy barriers occur at the steps of K_2_S_6_ to K_2_S_4_ for all three cathodes, which are identified as the rate-determining step (RDS) of the whole reduction process. The highest RDS energy barrier (1.748 eV) for NC/S explains the difficulty of the long-chain to short-chain KPSs conversion. With the catalytic effect of W_2_C nanocrystals, the lower RDS energy barrier (1.033 eV) endows W_2_C@NC/S with more pronounced solid-state KPSs reduction. As for W_SA_-W_2_C@NC/S, the lowest RDS energy barrier (0.943 eV) ensured the advantage in kinetics of both long-chain and short-chain conversion. This optimally balanced multi-step successive reduction process was consistent with the prominent peaks of both R_1,2_ and R_3_ displayed in the CV curves.

Regarding to the anodic scan, peaks of O_1_ and O_2_ can be distinguished for all three cathodes, which are ascribed to solid-state KPSs oxidation and long-chain KPSs oxidation, respectively. Of note, the O_3_ peaks distinguishable in the low scan rate (Fig. 3a) integrated into the O_2_ peak as scan rate increased, thereby being considered as one characteristic O_2_ peak. The response specific current of O_2_ peak for NC/S enhanced with increasing scan rate, suggesting the intrinsically facile oxidation of long-chain KPSs. The O_1_ peak is much lower than O_2_ peak for NC/S at high scan rate, which is different from that for W_SA_-W_2_C@NC/S and W_2_C@NC/S. This phenomenon indicates the fewer KPSs involving in solid-state conversion during charge for NC/S due to the absence of catalytic species. In the cases of W_2_C@NC/S, the O_1_ peak became more pronounced, suggesting the catalytic effect of W_2_C towards short-chain KPSs oxidation. W_SA_-W_2_C@NC/S delivered the highest specific currents for O_1,2_ peaks, highlighting the most favorable oxidation process from K_2_S to long-chain KPSs. The K_2_S_6_ based symmetric cell tests further proved the most facile long-chain oxidation for W_SA_-W_2_C@NC (Supplementary Fig. 29).

The K_2_S decomposition is the initial step of charging, which is also the most critical obstacle for efficient K-S electrochemistry in KSBs^18,26,27^. Therefore, the decomposition energy barriers of K_2_S on W_SA_-W_2_C@NC, W_2_C@NC and NC substrates were calculated to identify the reason for different charging behaviors of the cathodes (Fig. 4c). Based on the configuration determination of initial, transition and final states, the energy barriers for K_2_S decomposition are respectively 0.32, 0.49 and 0.97 eV for W_SA_-W_2_C@NC, W_2_C@NC and NC, suggesting the highest catalytic activity of W_SA_-W_2_C site towards K_2_S decomposition.

As the transition state provides a snapshot of the real-time K_2_S dissociation process, the characteristic of transition configuration reveals the origin of catalytic activity (Supplementary Fig. 30). The strength of W‒S bond between W_2_C and K_2_S derives from the *d*-*p* orbital hybridization^37,60^, which is an effective descriptor for the catalytic capability^18,35^. Basically, weaker W‒S bond strength would lead to more facile K_2_S decomposition and lower overpotential of K_2_S oxidation^18^. Projected crystal orbital Hamilton population (pCOHP) analysis were performed to evaluate the W‒S bond strength in the transition states of dynamic interaction between K_2_S and W_SA_-W_2_C, W_2_C sites (Fig. 4d). According to the integrated-COHP (ICOHP) values, the W‒S bond between K_2_S and W_SA_-W_2_C site is weaker due to the modulation of W_SA_ moieties^37,61,62^, suggesting the easier K_2_S dissociation stemming from the catalytic effect of W_SA_-W_2_C.

To experimentally evaluate the effect of different tungsten species towards the sulfur redox kinetics, the Tafel slopes and b values were extracted from multi-rate scan CV curves. The b value in *i* = a *v*^b^ reflects the reaction kinetics (*i* and *v* are the response specific current and scan rate)^63,64^. As shown in Fig. 4e, the b values of R_3_ (O_1_) peaks for W_SA_-W_2_C@NC/S, W_2_C@NC/S and NC/S were 0.878 (0.727), 0.831 (0.560) and 0.821 (0.518), respectively. The highest b values of R_3_/O_1_ for W_SA_-W_2_C@NC/S cathode indicate the most facile solid-state KPSs conversion, which is the essential reason for the highest sulfur utilization, optimal rate and cycling capability.

The redox kinetics associated with short-chain KPSs conversion can also be interpreted by the Tafel slopes. In Fig. 4f, both W_SA_-W_2_C@NC/S and W_2_C@NC/S exhibited earlier R_3_ onset potential and distinctly lower Tafel slope than NC/S (488, 490 vs. 559 mV dec^–^^1^), which is directly related to the accelerated reduction of solid KPSs by the tungsten species catalysis. In the initial oxidation process, W_SA_-W_2_C@NC/S provided a more positive onset potential and exhibited a lower Tafel slope of 418 mV dec^–^^1^ for the decomposition of solid short-chain KPSs (K_2_S_2_/K_2_S) compared to W_2_C@NC/S and NC/S.

The inferior Tafel slope of W_2_C@NC/S reveals the subtle difference in reaction mechanism caused by the participation of W_SA_. Due to the lower energy barrier for K_2_S_2_ migration on W_SA_ modified NC than that on W_SA_-free NC, the presence of W_SA_ facilitates the mass transfer around the W_2_C catalytic center, thereby maximizing the overall efficiency of K_2_S oxidation. Instead, the sluggish transportation of oxidation product could cause accumulation of insulting solid KPSs (e.g. K_2_S_2_) around the catalytic sites diminishing the activity for successive reaction (named catalytic poisoning)^65^. The W_SA_-W_2_C composite synergizing the catalytic conversion and facilitated migration towards KPSs is the key for the highly efficient solid-state KPSs conversion. Of note, W_2_C@NC/S and NC/S delivered comparable Tafel slopes near the O_1_ peaks, which is direct evidence of the poisoning effect on W_2_C catalytic site in W_2_C@NC/S.

The more facile KPSs conversion kinetics endowed by the synergy of W_SA_ and W_2_C can also be demonstrated by visual cell measurements. As shown in Fig. 4g, the visual cells employing potassium foils as anodes and W_SA_-W_2_C@NC/S, W_2_C@NC/S, NC/S as cathodes underwent 12 h standing and one entire discharging/charging cycle. The amount of KPSs dissolving in the electrolyte during this process is a reasonable descriptor for the KPSs conversion kinetics, because sluggish KPSs conversion provides more time for KPS dissolving. At the end point of charge-3.0 V, the electrolyte for W_SA_-W_2_C@NC/S visual cell remained colorless with negligible KPS signal detected in UV-vis spectrum (Fig. 4h). On the contrary, the electrolytes for W_2_C@NC/S and NC/S visual cells dissolved much more KPS, suggesting the less facile KPS conversion.

### Sulfur redox durability under synergy of KPS conversion and migration

**Fig. 5:**
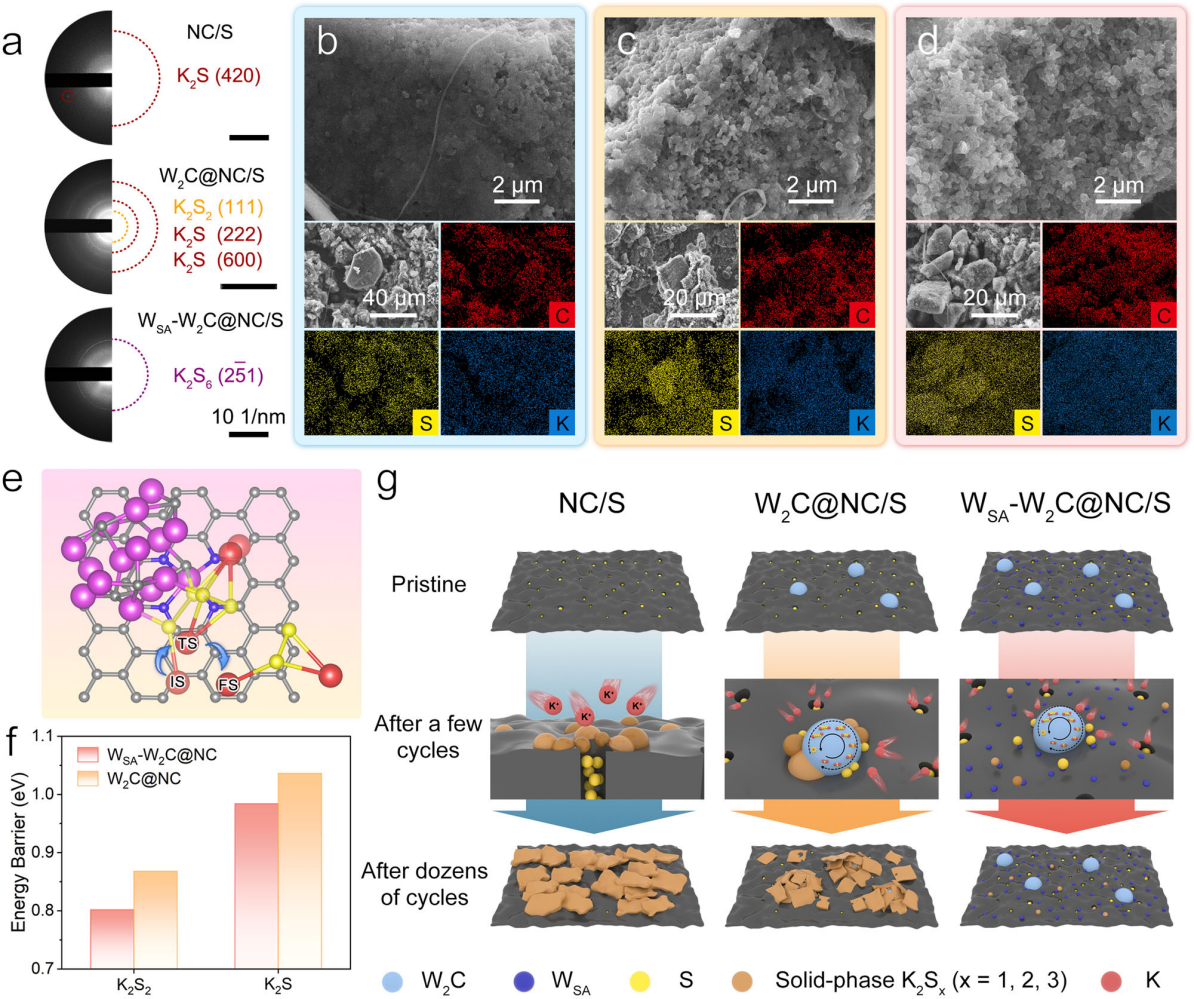
Investigations on sulfur redox durability in KSBs. **a** SAED patterns of post-cycled NC/S, W_2_C@NC/S, or W_SA_-W_2_C@NC/S cathodes. **b**–**d** SEM images and corresponding EDS mapping of post-cycled NC/S (**b**), W_2_C@NC/S (**c**) and W_SA_-W_2_C@NC/S (**d**) cathodes. **e** Configuration evolution of K_2_S_2_ migration from W_2_C catalytic site to W_SA_ modified NC substrate. **f** Energy barriers of K_2_S_2_ and K_2_S migration from W_2_C catalytic site to W_SA_ modified NC substrate or pure W_SA_-free NC substrate. **g** Schematic illustration of the potassium-sulfur electrochemistry in KSBs employing NC/S, W_2_C@NC/S and W_SA_-W_2_C@NC/S cathodes. Source data are provided as a Source Data file.

The analyses above revealed the decisive role of W_SA_-W_2_C composite in affecting the kinetics of solid-state KPSs conversion. To demonstrate the correlation between the solid-state KPSs conversion and cyclability of KSBs, the post-cycled cathodes (in charged states) were collected and examined. As shown in Fig. 5a and Supplementary Fig. 31, the majority phases of sulfur species are determined to be K_2_S for NC/S, K_2_S for W_2_C@NC/S, and K_2_S_6_ for W_SA_-W_2_C@NC/S. The result reveals that the sulfur species in NC/S and W_2_C@NC/S became mainly inert K_2_S after cycling, which cannot be oxidized back to provide reversible capacity.

The morphology and elemental distribution of the post-cycled cathodes were demonstrated in Fig. 5b-d. Severe agglomeration occurred in NC/S cathode, and the original polyhedrons can no longer be discerned, which is due to the extremely thick accumulation of sulfide cementing the polyhedrons^66,67^. The element mapping of sulfur, potassium and carbon reveals the large size (ca. 30 μm) potassium sulfide particles that detached from the carbon matrix. This phenomenon also appeared for post-cycled W_2_C@NC/S cathode (Fig. 5c). Although the polyhedron agglomeration is less severe, the non-uniform sulfur and potassium distribution reveal the segregation of potassium-rich sulfides in the cathode. As for post-cycled W_SA_-W_2_C@NC/S cathode, the polyhedron morphology almost remained unchanged (Fig. 5d). More importantly, the sulfur mapping overlapped with the shape of micro-size particles, but the potassium distributes uniformly all over the cathode, suggesting the absence of potassium-rich sulfide segregation.

The SEM/EDS results supplement the SAED analyses, further verifying that the accumulation of inert potassium sulfides is the essential reason causing the failure of W_2_C@NC/S and NC/S based KSBs. We claim that the facilitated migration of the potassium sulfide by W_SA_ plays a key role in significantly alleviating the sulfide accumulation. To prove this point, the migration behaviors of K_2_S_2_ and K_2_S from W_2_C catalytic site to the carbon substrate were investigated (Fig. 5e and Supplementary Fig. 32). The corresponding energy barriers for the migrations were summarized in Fig. 5f. Apparently, with the participation of W_SA_, the transfers of K_2_S_2_ and K_2_S from catalytic site can be accelerated due to the distinctly lower migration energy barrier. During the discharge, the fast detachment of K_2_S reduction product from catalytic site avoids the local accumulation of insulating K_2_S. In the charge, the oxidation product of K_2_S_2_ can efficiently diffuse from catalytic site to the substrate, alleviating the catalytic poisoning and maintaining the consecutive K_2_S decomposition. These effects become more crucial at high specific current densities and prolonged cycling, which endows W_SA_-W_2_C@NC with superior rate capability and long cycling stability.

## Discussion

The mechanisms of potassium-sulfur electrochemistry in the cathodes are demonstrated in Fig. 5g. Due to the absence of functional species in NC host, the sulfur redox kinetics is inferior especially for the solid-state KPSs conversion. Consequently, the confined sulfur species rapidly escape from the microporosity and formed a non-uniform deposition of inert K_2_S on host surface^68,69^. Upon repeated cycles, the K_2_S deposition would block the pores and cause thorough passivation. The continuous consumption of electrochemically active sulfur species leads to the fast failure of KSB, which occurs earlier at high rates. Although possessing W_2_C catalytic sites for W_2_C@NC host, the sluggish migration of solid KPSs like K_2_S/K_2_S_2_ would inevitably cause the catalytic poisoning due to the inefficiently prompt detachment of solid sulfides from the catalytic sites. In addition, the high energy barrier of KPSs migration on carbon substrate would restrict the contact between sulfur species and catalytic sites. Therefore, the accumulation of inert sulfides would continuously aggravate the cell performance upon prolonged cycling and finally lead to KSB failure^12,28^. With regard to W_SA_-W_2_C@NC/S cathode, the W_SA_-W_2_C composite synergizes the facilitated migration and catalytic conversion towards polysulfides. The facile migration of KPSs on W_SA_ modified carbon substrate addresses the mismatch in distribution between ubiquitous sulfur species and isolated catalytic sites. The accelerated transfer of solid K_2_S/K_2_S_2_ around W_2_C sites prevents the accumulation of inert sulfides and alleviates the catalytic poisoning. Resultantly, high sulfur utilization can be achieved and efficient sulfur redox can be maintained at the conditions of high rates and prolonged cycling, enabling the unprecedented reversible capacity, superior rate performance and long lifespan of KSBs.

In summary, we adopted the strategy of synergizing the facilitated KPSs migration and catalytic KPSs conversion for KSBs. Based on the theoretical screening and sophisticated synthesis procedure, NC equipped with W_SA_-W_2_C composite was fabricated as sulfur hosts. Comprehensive experimental analyses and theoretical calculations revealed that W_SA_ species optimize the catalytic capability of W_2_C species for solid-state KPSs conversion (more facile K_2_S dissociation) and accelerate the KPSs migration near catalytic sites (lower energy barrier for K_2_S/K_2_S_2_ diffusion). As a result, the insulating sulfides accumulation and catalytic poisoning were effectively alleviated in W_SA_-W_2_C@NC/S cathode than the W_SA_ free W_2_C@NC/S and tungsten free NC/S counterparts, which contributes to the ultra-high sulfur utilization, efficient sulfur redox at high rate and long cycling stability. The validated strategy of KPSs migration acceleration and conversion catalysis bi-functional sulfur host brings more possibility for developing high performance KSBs.

## Methods

### Chemicals

Sodium tungstate dihydrate (Na_2_WO_4_·2H_2_O, 99%) and 2-Methylimidazole (2-MIM, 98%), zinc acetate (Zn(CH_3_COO)_2_·2H_2_O, 99.98%) were purchased from HEOWNS. Methanol (99.9%) was purchased from Shanghai Macklin Biochemical Co., Ltd. N, N-Dimethylformamide (DMF, 99.5%) was purchased from Tianjin Kemiou Chemical Reagent Co., Ltd. Hydrochloric acid (HCl, 36.0-38.0 wt.%) was purchased from Rionion Development Co., Ltd. Sulfur was purchased from Sigma-Aldrich. 0.8 M KPF_6_ in EC/DEC was purchased from Suzhou DoDoChem Co., Ltd. N-methyl−2-pyrrolidone (NMP) and poly(vinylidene fluoride) (PVDF) were purchased from Guangdong Canrd New Energy Technology Co., Ltd. Multiwalled carbon nanotubes (MWCNTs) was purchased from Nanjing XFNANO Materials Tech Co., Ltd. Potassium metal was purchased from Alfa Aesar (China) Chemicals Co., Ltd.

### Synthesis of ZIF-8 and WO_4_^2-^ modified ZIF-8 (WO_4_^2-^-ZIF-8)

In a typical procedure, 8.95 g (0.109 mol) 2 MIM and 2.39 g (10.9 mmol) Zn(CH_3_COO)_2_·2H_2_O were dissolved in 40 ml of deionized water under continuous magnetic stirring and labeled as solution A and solution B, respectively. Subsequently, solution B was rapidly poured into solution A and aged at room temperature for 48 h. The white ZIF-8 powder was collected by centrifugation, washed three times with methanol, and then dried overnight in a vacuum oven at 60 °C. 0.40 g dried ZIF-8 powder was dispersed in 60 ml DMF solution, and 0.20 g (0.606 mmol) Na_2_WO_4_·2H_2_O was added afterwards. The solution was kept stirring until became homogeneous. The mixed solution was then transferred to a 100 ml Teflon-lined stainless-steel autoclave and underwent a solvothermal process at 150 °C for 12 h. After cooling to room temperature, the light yellow WO_4_^2-^-ZIF-8 powder was obtained by centrifugation, washing with deionized water and methanol alternately three times, and drying at 60 °C.

### Synthesis of W_SA_-W_2_C@NC

The WO_4_^2-^-ZIF-8 powder was heated to 400 °C and kept for 2 h under N_2_ atmosphere with a slow heating rate of 2 °C min^−1^ to prevent the formation of large compound particles during the annealing process. Subsequently, the temperature was increased to 800 °C at a rate of 2 °C min^−1^ and kept for another 2 h. The obtained powders were dispersed in 50 ml 6 M HCl and held at 60 °C for 4 h to etch off the Zn species and to generate microporous. Afterward, the powder was washed with plenty of deionized water and dried in a vacuum oven at 60 °C overnight to obtain the product labeled as W_SA_-W_2_C@NC.

### Synthesis of W_2_C@NC

0.20 g (0.606 mmol) Na_2_WO_4_·2H_2_O and 0.40 g ZIF-8 powder was mixed and grounded in the mortar for 30 min. Afterward, the homogeneous mixture was heated up to 400 °C and kept for 2 h under N_2_ atmosphere with a slow heating rate of 2 °C min^–^^1^. Then, the nitrogen-filled tube furnace was ramped up to 800 °C at the same heating rate and kept for another 2 h. Afterwards, the residual Zn species in the as-prepared powder was further etched by 6 M HCl and held at 60 °C for 4 h. After collected by infiltration, the powder was washed with plenty of deionized water and dried in a vacuum oven at 60 °C overnight. The obtained product was labeled as W_2_C@NC.

### Synthesis of NC

The ZIF-8 powder was directly carbonized according to the same two-step heating procedure. In a typical process, ZIF-8 powders were filled in a porcelain boat and heated up to 400 °C for 2 h in a nitrogen-filled tube furnace with a heating rate of 2 °C min^−1^, and then to 800 °C for another 2 h. Then, the as-prepared powder was treated with 6 M hydrochloric acid solution kept at 60 °C for 4 h, followed by washing with a large amount of deionized water and then placed in a vacuum oven at 60 °C overnight. The obtained product was labeled as NC.

### Synthesis of W_SA_-W_2_C-H@NC and W_SA_-W_2_C-L@NC

The synthesis of W_SA_-W_2_C-H@NC and W_SA_-W_2_C-L@NC followed the same procedure of W_SA_-W_2_C@NC except different amount of Na_2_WO_4_·2H_2_O (0.60 g (1.819 mmol) for W_SA_-W_2_C-H@NC and 0.10 g (0.303 mmol) for W_SA_-W_2_C-L@NC) were added for preparing WO_4_^2-^-ZIF-8 precursors.

### Synthesis of W_SA_-W_2_C@NC/S, W_2_C@NC/S and NC/S composites

The as-prepared W_SA_-W_2_C@NC, W_2_C@NC and NC hosts were uniformly mixed with sulfur powder with a mass ratio of 1:1 and sealed in a glass tube under vacuum. The glass tube was heated to 155 °C at a rate of 5 °C min^−1^ and kept for 24 h, followed by heating to 300 °C and holding for 2 h. Then, the obtained powders were heated at 200 °C for 1 h under flowing Ar in a tube furnace. The products are termed as W_SA_-W_2_C@NC/S (or NC/S, W_2_C@NC/S) composite.

### Adsorption measurements

The K_2_S_6_ solution for adsorption measurements was prepared by mixing potassium sulfide (K_2_S) and sulfur with a molar ratio of 1:5 in dimethyl ether (DME). 5 mg W_SA_-W_2_C@NC, W_2_C@NC, and NC were added into the 2 ml 0.02 M K_2_S_6_ solution, respectively, with the blank K_2_S_6_ solution as a reference.

### Materials characterization

The scanning electron microscopy (SEM) images were obtained by JEOL JSM-7800F with an energy dispersive spectrometer (EDS). The transmission electron microscopy (TEM) images and selected area electron diffraction (SAED) patterns were recorded by JEOL JEM-F200. The aberration-corrected high angle annular dark-field scanning transmission electron microscopy (HAADF-STEM) images were collected by JEOL JEM-ARM200F. The X-ray photoelectron spectroscopy (XPS) analysis was performed on Kratos Axis Ultra DLD spectrometer. Raman spectra were recorded with Horiba LabRam HR Evolution spectrometer. Nitrogen adsorption-desorption isotherm measurement was conducted using Micromeritics ASAP 2460 system. The thermogravimetric analysis (TGA) was investigated by using NETZSCH STA 449 C instrument. The ultraviolet-visible (UV-vis) spectra were performed by Shimadzu UV-2700 spectrophotometer. The in-situ X-ray diffraction (XRD) patterns were performed using Bruker D8 Advance with Cu Kα radiation (λ = 0.15406 nm) at 40 kV and 40 mA. The X-ray absorption structure (XAS) spectra (W *L*-edge) were measured in Shanghai Synchrotron Radiation Facility (SSRF).

### Electrochemical measurements

Electrochemical tests were performed by assembling CR2032-type coin cells in an Argon-filled glove box. 60 wt.% cathode materials (W_SA_-W_2_C@NC/S (40.56 wt.% sulfur), W_2_C@NC/S (37.91 wt.% sulfur) or NC/S (43.93 wt.% sulfur)), 30 wt.% MWCNTs, and 10 wt.% PVDF were mixed with an appropriate amount of NMP for the preparation of a uniform slurry. The obtained slurry was cast on carbon-coated aluminum foil with the thickness of 250 μm and dried overnight in a vacuum oven at 60 °C. The electrodes were punched into 12 mm diameter disks for battery assembly. Glass fiber (GF/C, Whatman) with the diameter of 19 mm was chosen as the separator. Potassium metal foils produced from surface oxide layer-free potassium blocks were directly applied as anode. The electrolyte is 0.8 M KPF_6_ in EC/DEC with a volume ratio of 1:1. The typical sulfur loading of the electrode is ca. 1 mg cm^–^^2^. Electrodes with high sulfur loading of ca. 2.8 and ca. 3.6 mg cm^–^^2^ were also prepared for electrochemical tests. For the full wetting of the glass fiber separator, the normal electrolyte dosage in one coin cell is 100 μL. Electrochemical measurements were performed on a NEWARE battery testing system and Solartron multi-channel electrochemical workstation with the voltage range of 0.5-3.0 V (vs. K^+^/K) in a thermostat at room temperature (25 °C). Cycling performance was conducted at specific currents of 167.5, 837.5, and 1675 mA g^−1^, and rate performance was conducted at a series of specific currents in the order of 167.5, 335, 502.5, 837.5, 1675, 3350, 5025, and 167.5 mA g^−1^, respectively.

### Symmetric cell measurements

The K_2_S_6_ electrolyte was fabricated by adding K_2_S and sulfur (molar ratio corresponds to the nominal stoichiometry of K_2_S_6_) into the tetra ethylene glycol dimethyl ether (TEGDME) with 3 M potassiumbis(trifluor-omethylsulfonyl)imide (KTFSI), and then stirring at 60 °C for 24 h. 100 μL obtained K_2_S_6_-contained electrolyte (0.052 M) with the identical anodes and cathodes of W_SA_-W_2_C@NC, W_2_C@NC, and NC were assembled into the symmetric cells for the polysulfides conversion mechanism study.

### Visual cell measurements

The cathode materials (W_SA_-W_2_C@NC/S, W_2_C@NC/S, or NC/S), MWCNTs, PVDF with a mass ratio of 6:3:1 was dispersed in NMP, and coated on carbon paper, followed by drying at 60 °C for overnight. The potassium foil that was stamped onto the stainless-steel collector was used as anode. Both anode and cathode were clamped by alligator clips, and the electrolyte is 4 M KTFSI in DME. Finally, the reaction vessels were sealed for galvanostatic charge-discharge measurements at 167.5 mA g^–^^1^ on Solartron multi-channel electrochemical workstation.

### Computational methods

Details of the computational methods is provided within the Supplementary Information.

### Supplementary information

Supplementary Information

Peer Review File

### Source data

Source Data

## Data Availability

The data generated in this study are provided in the Supplementary Information and are available from the authors upon request. Source data are provided with this paper.
