# Supplementary material for: Optimizing potassium polysulfides for high performance potassium-sulfur batteries
Source: Nat Commun. 2024 Feb 2;15:1005. doi: 10.1038/s41467-024-45405-w (PMC10837207; doi:10.1038/s41467-024-45405-w)
Supplement: Supplementary file 1 — Supplementary Information [file 41467_2024_45405_MOESM1_ESM.pdf]

Supplementary Information

Optimizing potassium polysulfides for high performance potassium-sulfur batteries

Wanqing Song[1], Xinyi Yang[1], Tao Zhang[1], Zechuan Huang[1], Haozhi Wang[1,2], Jie Sun[3], Yunhua Xu[1], Jia Ding[1]*, Wenbin Hu[1,4]**

[1]School of Materials Science and Engineering, Tianjin Key Laboratory of Composite and Functional Materials Key Laboratory of Advanced Ceramics and Machining Technology (Ministry of Education), Tianjin University, Tianjin, China.
[2]School of Materials Science and Engineering, Hainan University, Haikou, China.
[3]School of Chemical Engineering and Technology, Tianjin University, Tianjin, China.
[4]Joint School of National University of Singapore and Tianjin University, International Campus of Tianjin University, Binhai New City, Fuzhou, China.
*Email: jiading@tju.edu.cn; wbhu@tju.edu.cn; hzwang001@tju.edu.cn

Experimental Section

Computational methods

All the calculations were performed within the framework of the density functional theory (DFT) as implemented in the Vienna Ab initio Software Package (VASP 5.4.4) code within the Perdew–Burke–Ernzerhof (PBE) generalized gradient approximation and the projected augmented wave (PAW) method.[1-4] The cutoff energy for the plane-wave basis set was set to 400 eV. W (110), W_2C (102), WC (101), WN (112), WO_3 (100), WS_2 (002), and WSe_2 (006) surfaces were constructed to model the catalysts in this work. A vacuum layer of 15 Å was introduced to avoid interactions between periodic images. The Brillouin zone of the surface unit cell was sampled by Monkhorst–Pack (MP) grids, with a different k-point meshs for catalysts optimizations.[5] The k-point mesh density of $2\pi \times 0.04$ Å^{-1} was used for structures optimizations. The convergence criterion for the electronic self-consistent iteration and force was set to 10^{-5} eV and 0.01 eV Å^{-1}, respectively. The climbing image nudged elastic band (CI-NEB)[6-8] method was used to confirm the transition states with only one imaginary frequency along the reaction coordinates. The adsorption energy (E_{ads}) of the surface species is defined by

$$E_{ads} = E_{total} - E_{surface} - E_{species} \quad (1)$$

where E_{total} represents the total energy of the adsorbed species with catalyst surface, $E_{surface}$ is the energy of the empty surface, and $E_{species}$ is the energy of the species in the gas phase.

The activation barrier (E_a) and reaction energy (E_r) are defined by

$$E_a = E_{TS} - E_{IS} \quad (2)$$

$$E_r = E_{FS} - E_{IS} \quad (3)$$

where E_{TS} represents the energy of transition state, E_{IS} represents the energy of initial state, and E_{FS} represents the energy of final state.

Surface free energy, which is defined as the reversible work per unit area needed to create a new surface out of the bulk, is calculated as:[9]

$$\gamma = \frac{G_{slab} - NG_{bulk}}{2A} \quad (4)$$

where G_{slab} and G_{bulk} are the Gibbs free energy of a slab and the Gibbs free energy per bulk atom, and A is the area of the exposed surface the slab.

Supplementary Figures and Tables

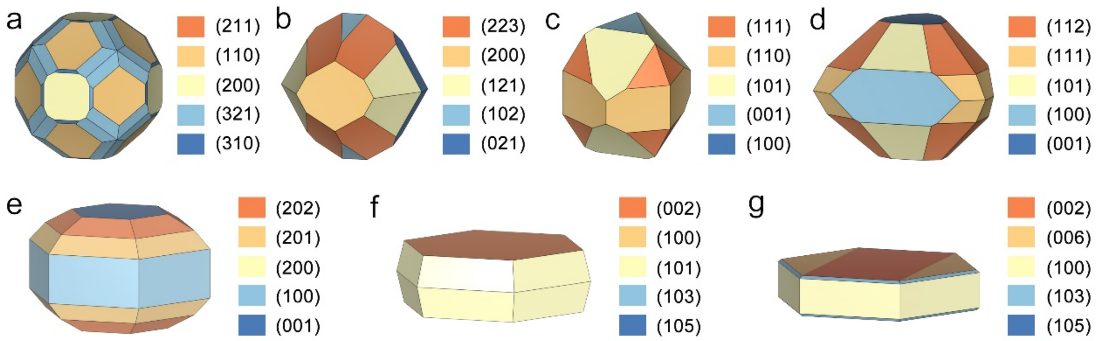

Supplementary Figure 1. Wulff construction of tungsten-based compounds. Equilibrium morphologies of **a** W metal, **b** W_2C, **c** WC, **d** WN, **e** WO_3, **f** WS_2 and **g** WSe_2 based on Wulff construction from DFT.

Supplementary Table 1. The surface energies of tungsten-based composite calculated with DFT.

Facet	γ (J m^{-2})	γ_i/γ_{total}	Facet	γ (J m^{-2})	γ_i/γ_{total}
W (110)	4.03	41.36%	W_2C (021)	4.04	8.50%
W (200)	4.15	18.95%	W_2C (102)	4.07	4.42%
W (211)	4.44	--	W_2C (121)	3.84	31.80%
W (310)	4.39	2.87%	W_2C (200)	3.91	13.04%
W (321)	4.29	36.82%	W_2C (223)	3.74	42.24%
Facet	γ (J m^{-2})	γ_i/γ_{total}	Facet	γ (J m^{-2})	γ_i/γ_{total}
WC (001)	5.55	4.87%	WN (001)	2.35	8.48%
WC (100)	7.82	--	WN (100)	2.23	22.90%
WC (101)	4.64	40.82%	WN (101)	2.29	23.41%
WC (110)	4.59	32.81%	WN (111)	2.77	13.86%
WC (111)	4.98	21.49%	WN (112)	2.47	31.34%
Facet	γ (J m^{-2})	γ_i/γ_{total}	Facet	γ (J m^{-2})	γ_i/γ_{total}
WO_3 (001)	0.49	15.05%	WS_2 (002)	0.24	55.00%
WO_3 (100)	0.70	32.43%	WS_2 (100)	1.53	--
WO_3 (200)	0.76	--	WS_2 (101)	0.66	45.00%
WO_3 (201)	0.67	27.43%	WS_2 (103)	1.27	--
WO_3 (202)	0.61	25.08%	WS_2 (105)	1.06	--
Facet	γ (J m^{-2})	γ_i/γ_{total}			
WSe_2 (002)	0.27	--			
WSe_2 (006)	0.27	66.37%			
WSe_2 (100)	1.19	27.38%			
WSe_2 (103)	1.19	--			
WSe_2 (105)	0.97	6.25%			

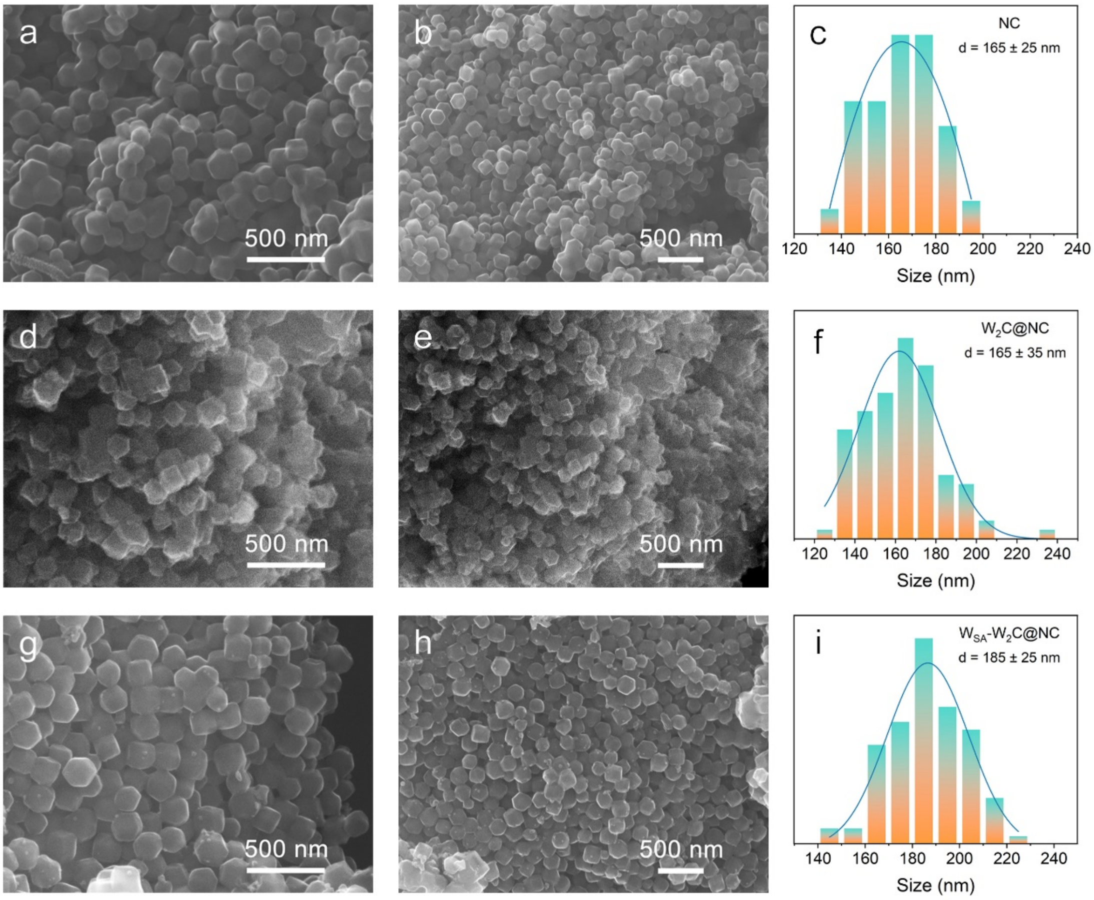

Supplementary Figure 2. Morphologies of sulfur hosts. SEM images and corresponding particle size distribution of **a-c** NC, **d-f** W_2C@NC, and **g-i** W_{SA}-W_2C@NC.

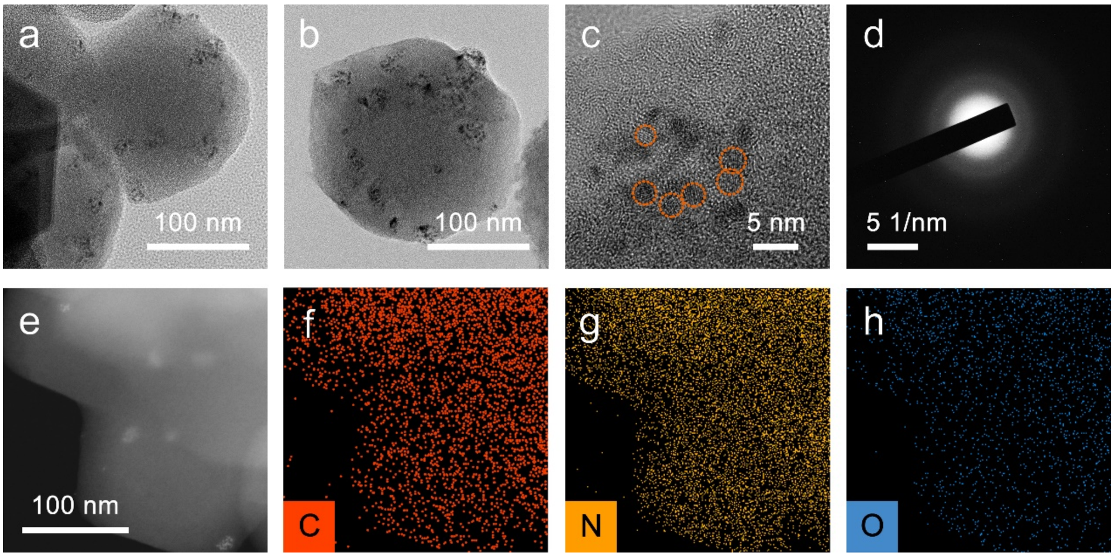

Supplementary Figure 3. Morphologies and elemental distribution of W_{SA}-W_2C@NC. a, b TEM images, **c** HRTEM image (W_2C nanocrystals are highlighted with orange dashed circles), **d** SAED image, **e** HAADF-STEM image and **f-h** corresponding EDS elemental maps of W_{SA}-W_2C@NC.

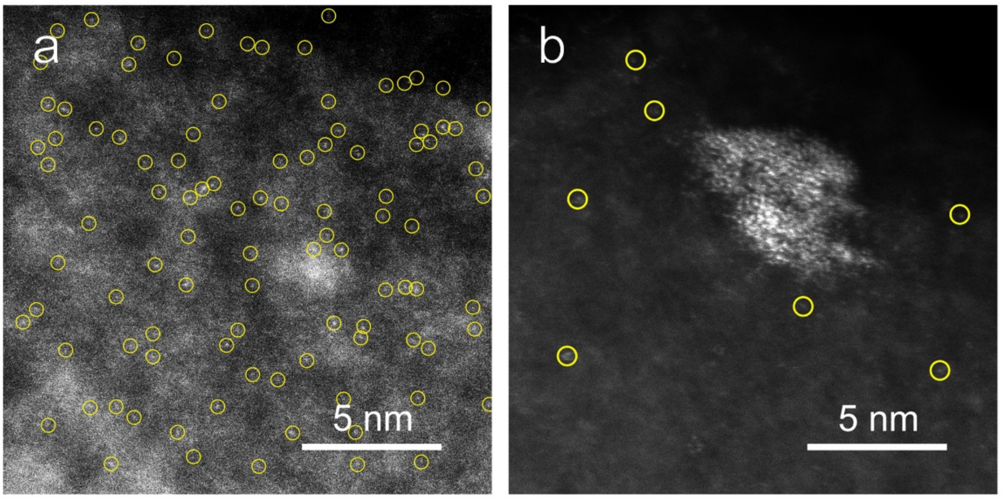

Supplementary Figure 4. Atomic investigation of W_{SA}-W_2C@NC and W_2C@NC. HAADF-STEM images of **a** W_{SA}-W_2C@NC and **b** W_2C@NC.

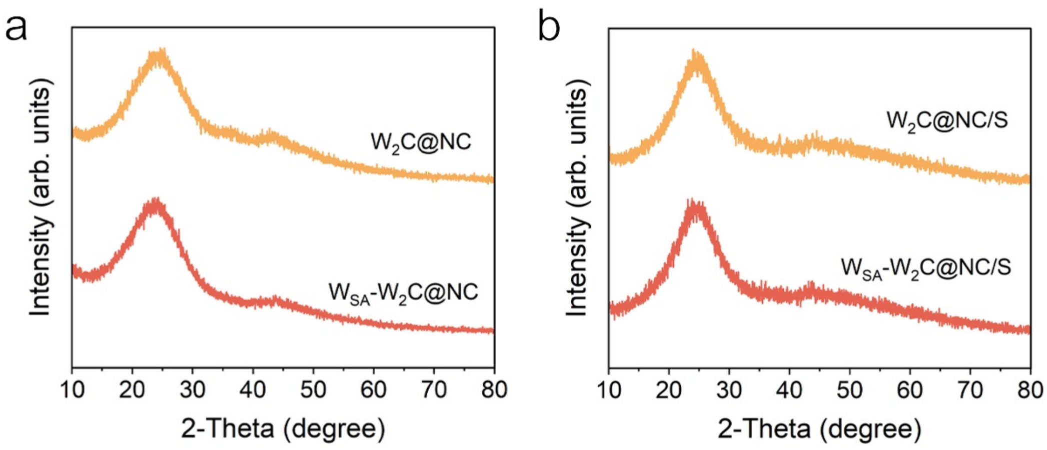

Supplementary Figure 5. Phase analysis of the hosts and the composites. XRD patterns of **a** W_{SA}-W_2C@NC and W_2C@NC, **b** W_{SA}-W_2C@NC/S and W_2C@NC/S.

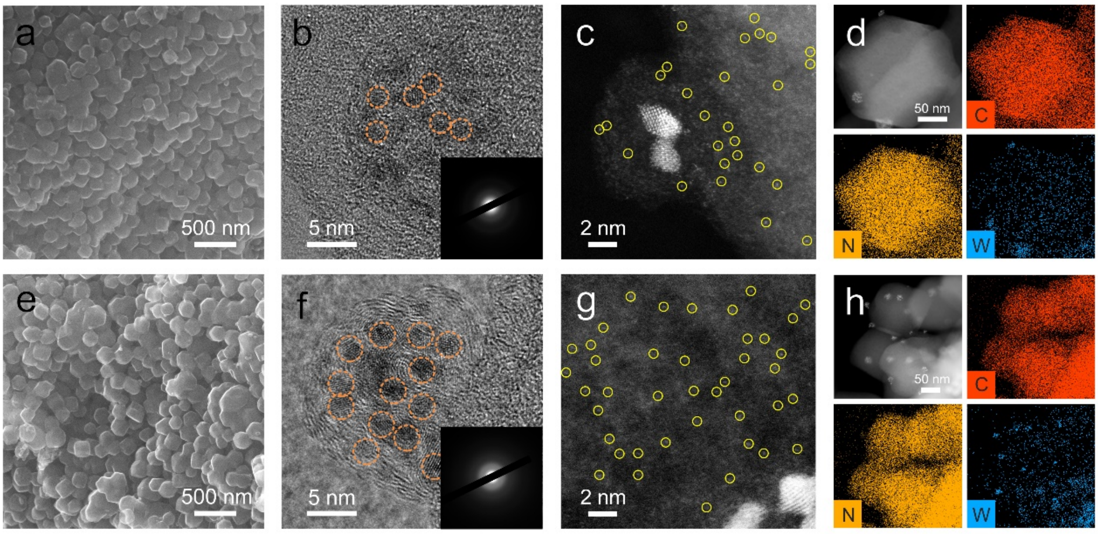

Supplementary Figure 6. Characterization of W_{SA}-W_2C-H&L@NC. a SEM image, **b** HRTEM image, **c** HADDF-STEM image and **d** EDS mapping of W_{SA}-W_2C-L@NC. **e** SEM image, **f** HRTEM image, **g** HADDF-STEM image and **h** EDS mapping of W_{SA}-W_2C-H@NC.

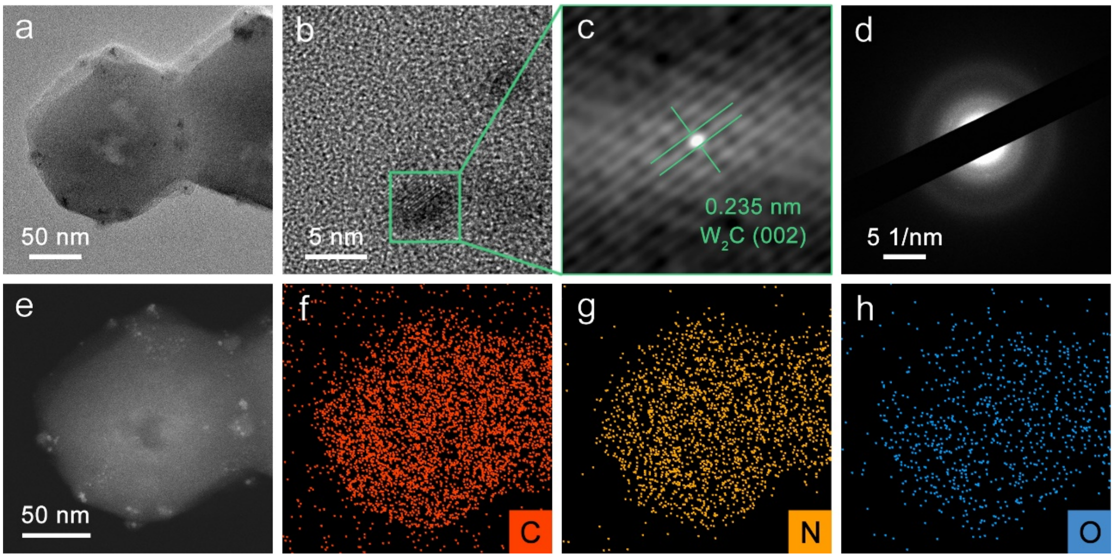

Supplementary Figure 7. Morphologies and elemental distribution of W_2C@NC. **a** TEM image, **b, c** HRTEM image with selected region autocorrelation image, **d** SAED image, **e** HAADF-STEM image and **f-h** corresponding EDS elemental maps of W_2C@NC.

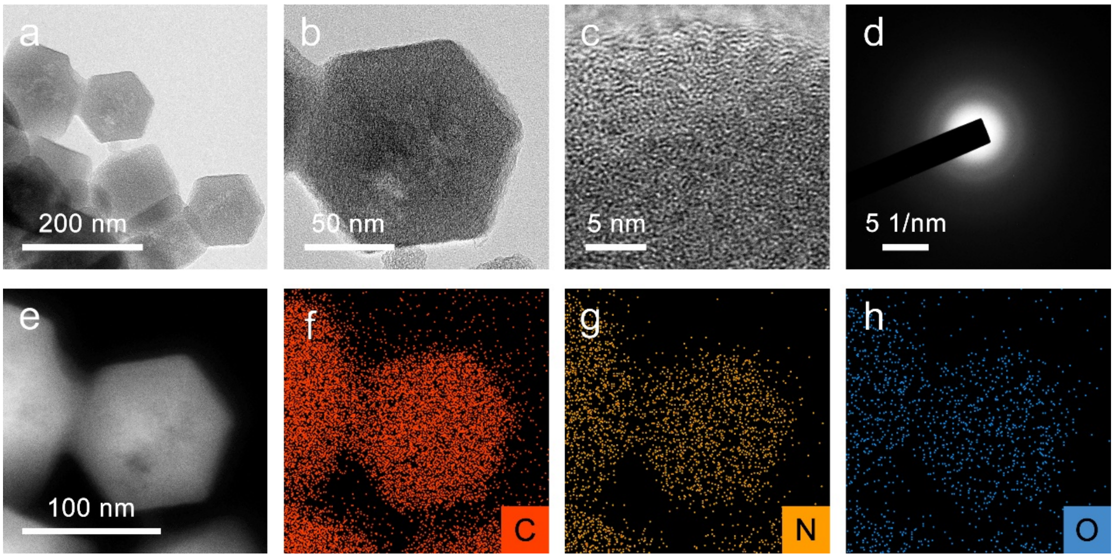

Supplementary Figure 8. Morphologies and element distribution of NC. a, b TEM images, **c** HRTEM image, **d** SAED image, **e** HAADF-STEM image and **f-h** corresponding EDS elemental maps of NC.

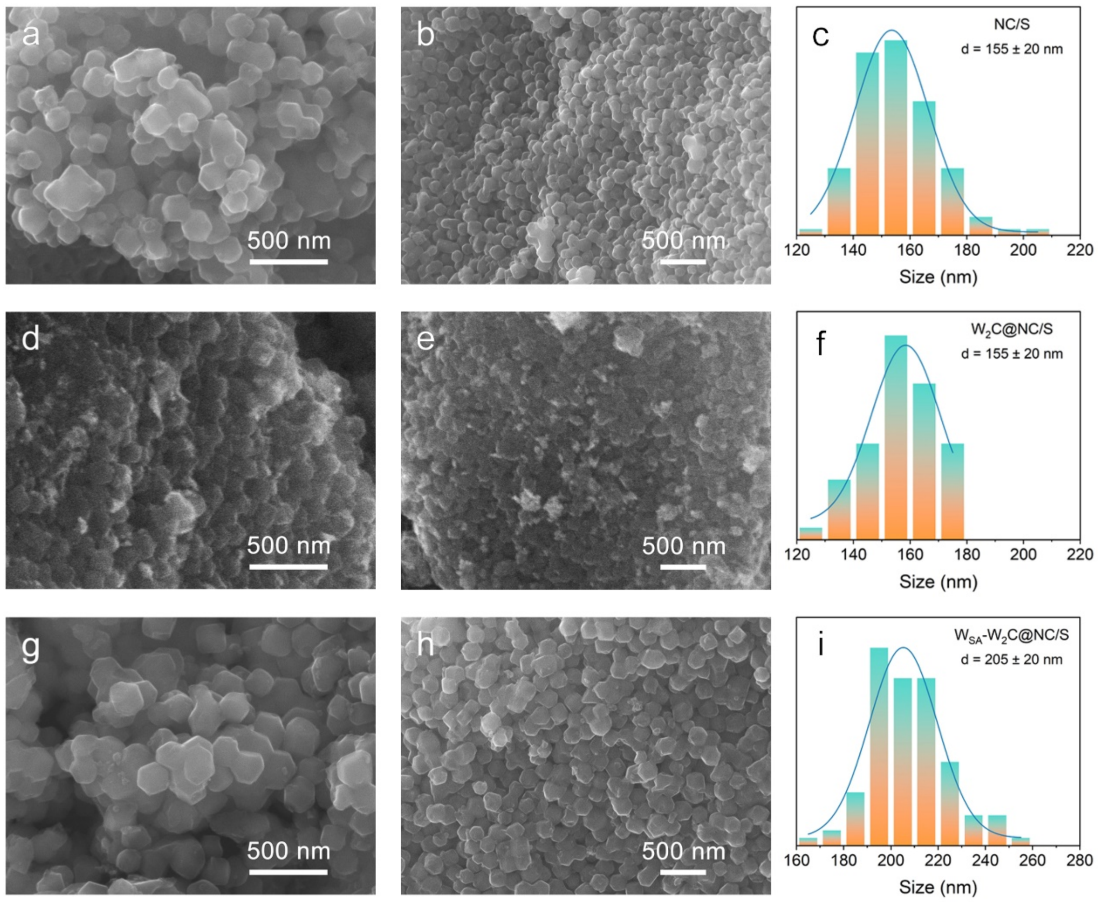

Supplementary Figure 9. Morphologies of the composites. SEM images and corresponding particle size distribution of **a-c** NC/S, **d-f** W_2C@NC/S, and **g-i** W_{SA}-W_2C@NC/S.

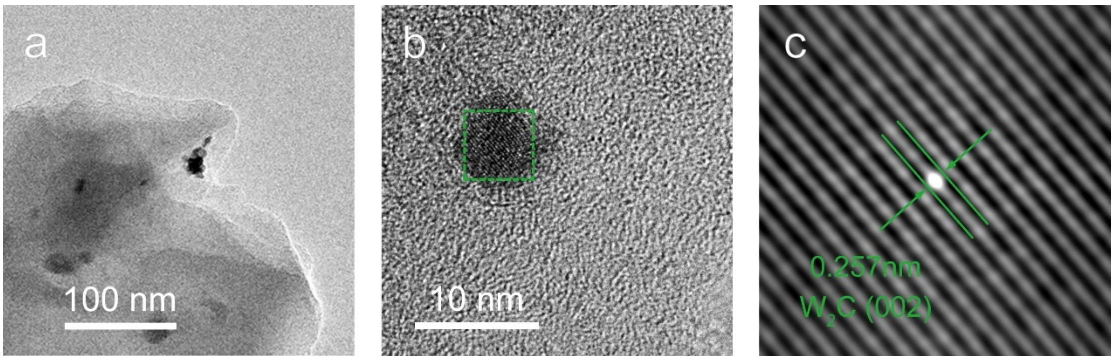

Supplementary Figure 10. Phase analysis of nanocrystals loaded on W_2C@NC/S. **a** TEM image, **b, c** HRTEM image with selected region autocorrelation image of W_2C@NC/S.

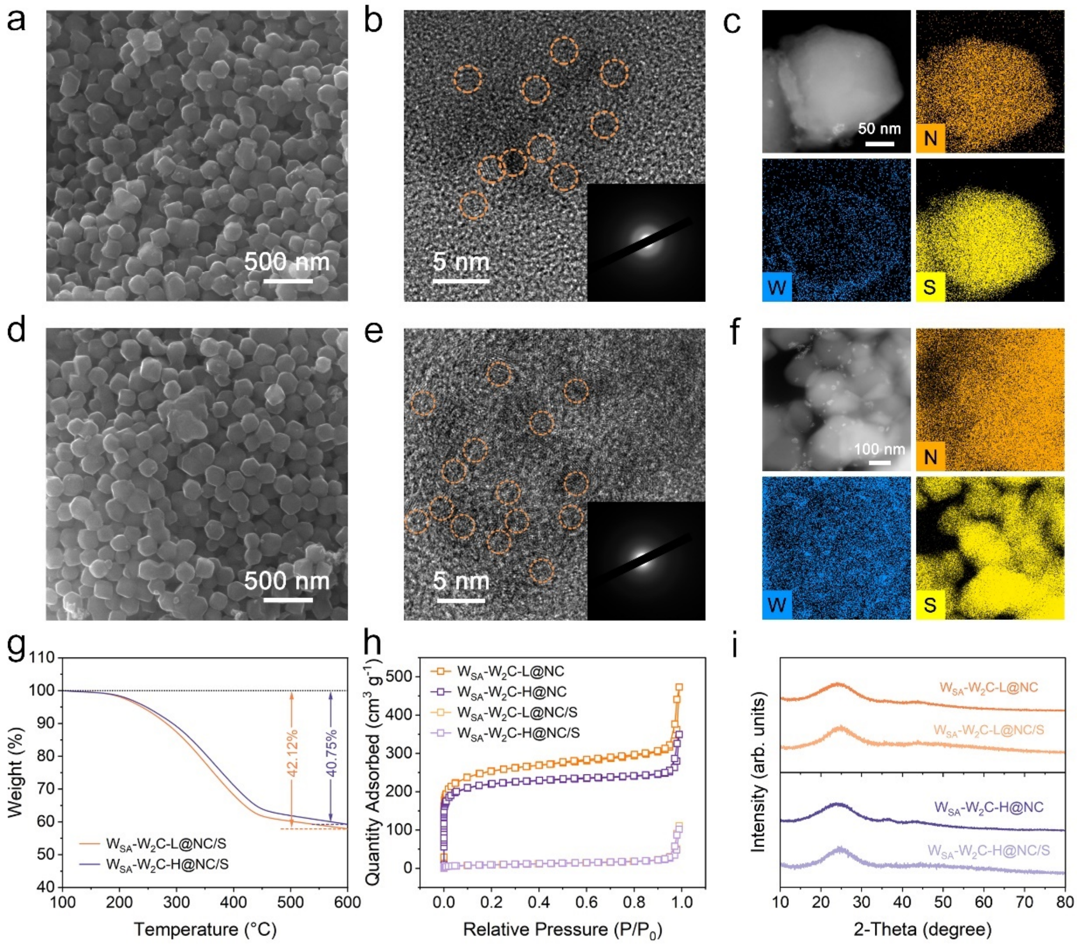

Supplementary Figure 11. Characterization of W_{SA}-W_2C-H&L@NC and W_{SA}-W_2C-H&L@NC/S. **a** SEM image, **b** HRTEM image, and **c** EDS mapping of W_{SA}-W_2C-L@NC/S. **d** SEM image, **e** HRTEM image, and **f** EDS mapping of W_{SA}-W_2C-H@NC/S. **g** TGA curves of W_{SA}-W_2C-L@NC/S and W_{SA}-W_2C-H@NC/S. **h** Nitrogen adsorption-desorption isotherms and **i** XRD patterns of W_{SA}-W_2C-L@NC, W_{SA}-W_2C-H@NC, W_{SA}-W_2C-L@NC/S, and W_{SA}-W_2C-H@NC/S.

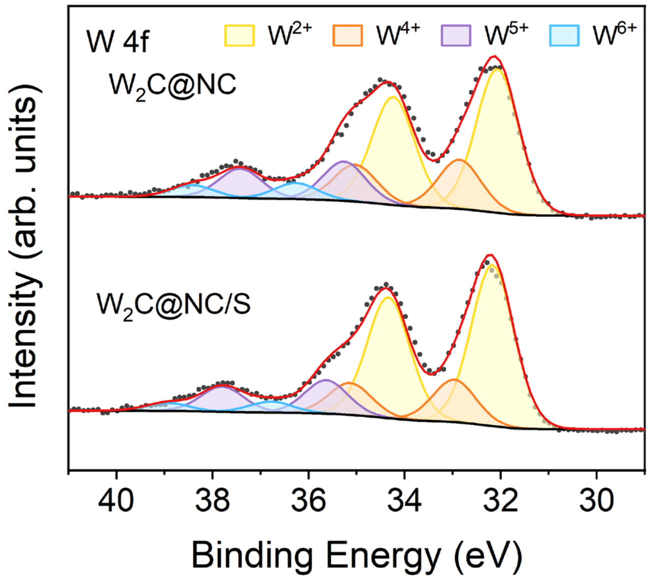

Supplementary Figure 12. Valence analysis of W_2C@NC and W_2C@NC/S. High-resolution W 4f XPS spectra for W_2C@NC and W_2C@NC/S.

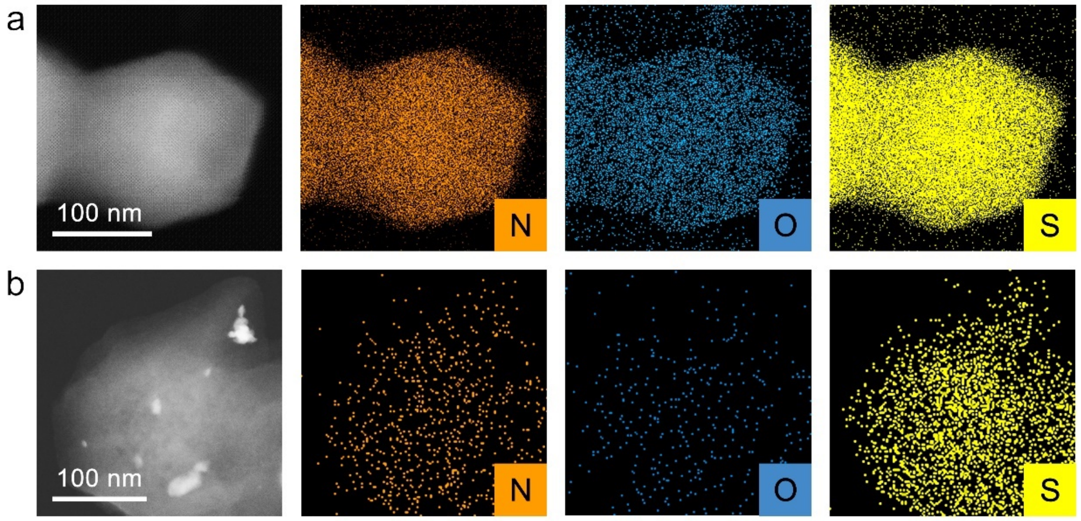

Supplementary Figure 13. Morphology and element distribution of NC/S and W_2C@NC/S. HAADF-STEM images and corresponding EDS elemental maps of **a** NC/S and **b** W_2C@NC/S.

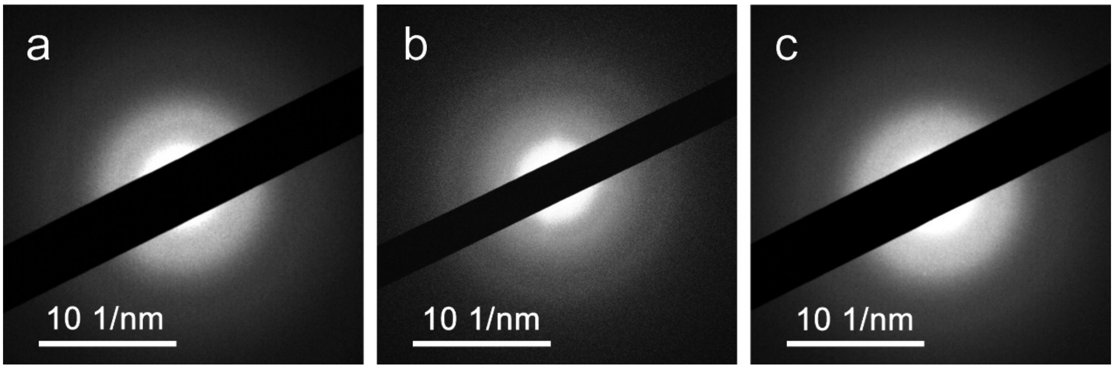

Supplementary Figure 14. Structural analysis of the composites. SAED patterns of **a** NC/S, **b** W_2C@NC/S, and **c** W_{SA}-W_2C@NC/S.

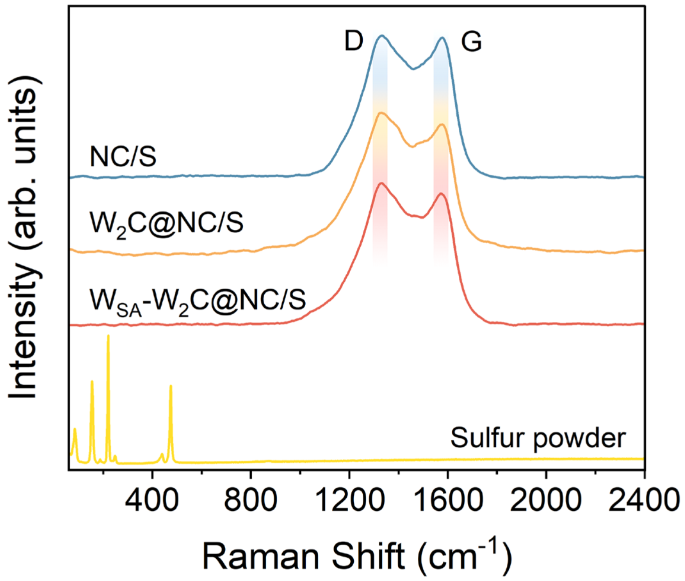

Supplementary Figure 15. Structural analysis of sulfur in the composites. Raman spectra of sulfur powder, NC/S, W_2C@NC/S and W_{SA}-W_2C@NC/S.

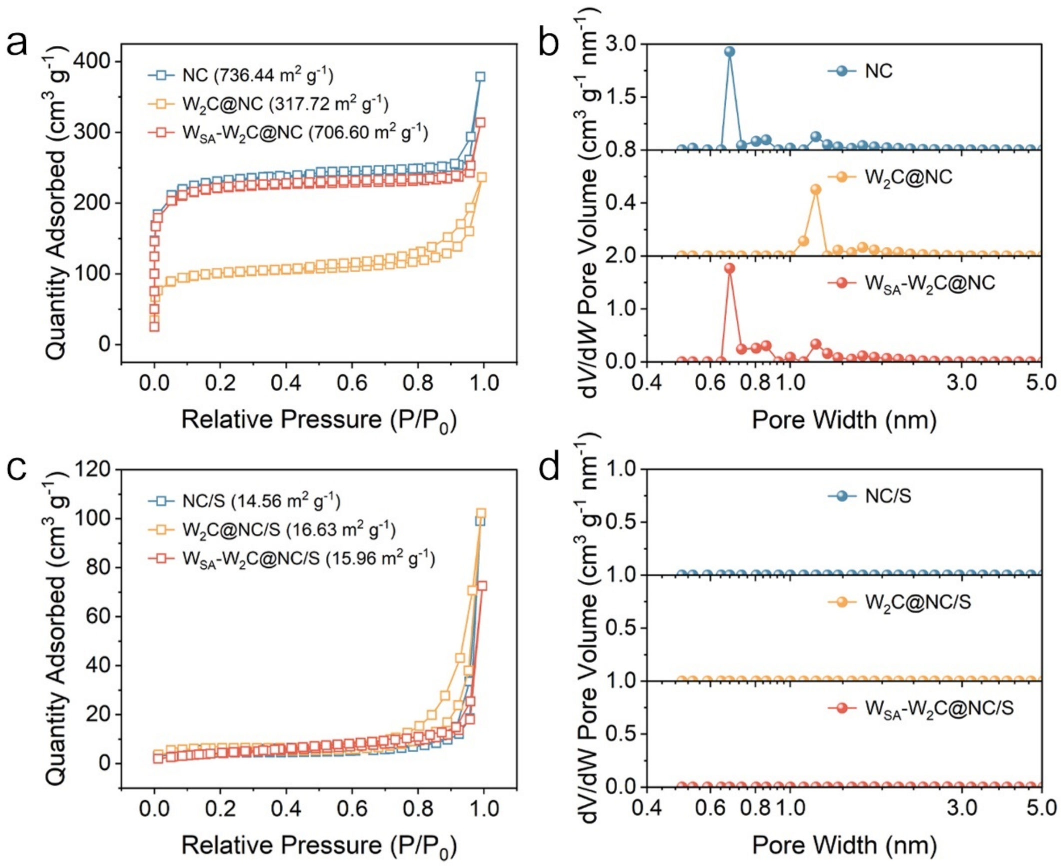

Supplementary Figure 16. Pore structure analysis of the hosts and the composites. a Nitrogen adsorption-desorption isotherms and **b** pore size distribution of NC, W_2C@NC and W_{SA}-W_2C@NC. **c** Nitrogen adsorption-desorption isotherms and **d** pore size distribution of NC/S, W_2C@NC/S and W_{SA}-W_2C@NC/S. BET surface values are annotated after the sample IDs.

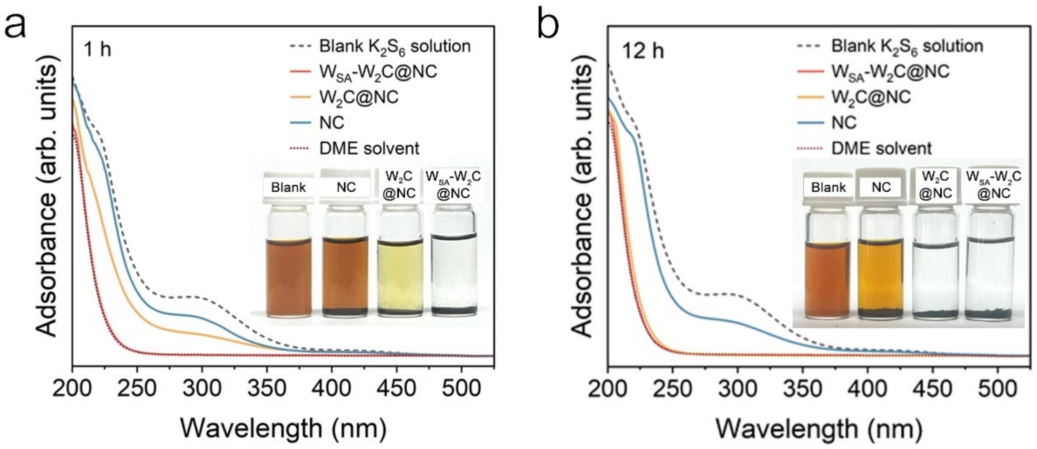

Supplementary Figure 17. K_2S_6 adsorption measurements of sulfur hosts. UV-vis spectra and the optical photos (inset) of different K_2S_6 solutions with different host materials immersed after **a** 1 h and **b** 12 h. Spectrum of pure DME solvent is also included for comparison.

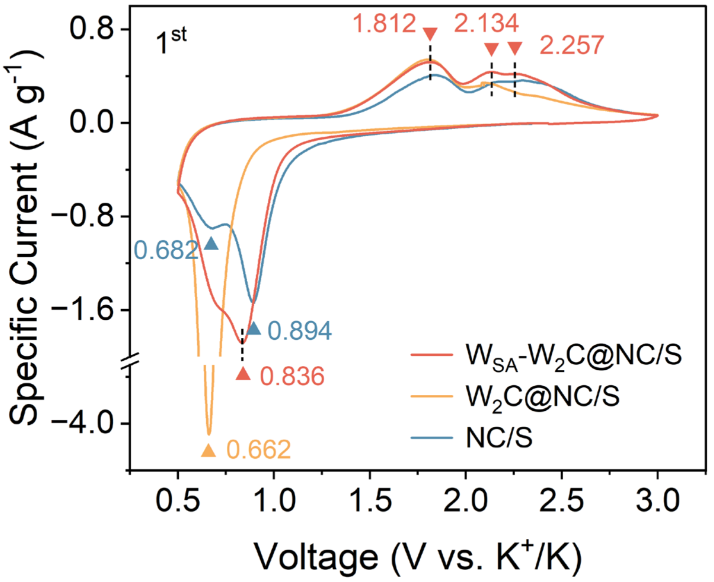

Supplementary Figure 18. CV curves of KSBs with different cathodes for the 1st cycle. CV curves of KSBs employing W_{SA}-W_2C@NC/S, W_2C@NC/S, and NC/S cathodes for the first cycle at 0.1 mV s^{-1}.

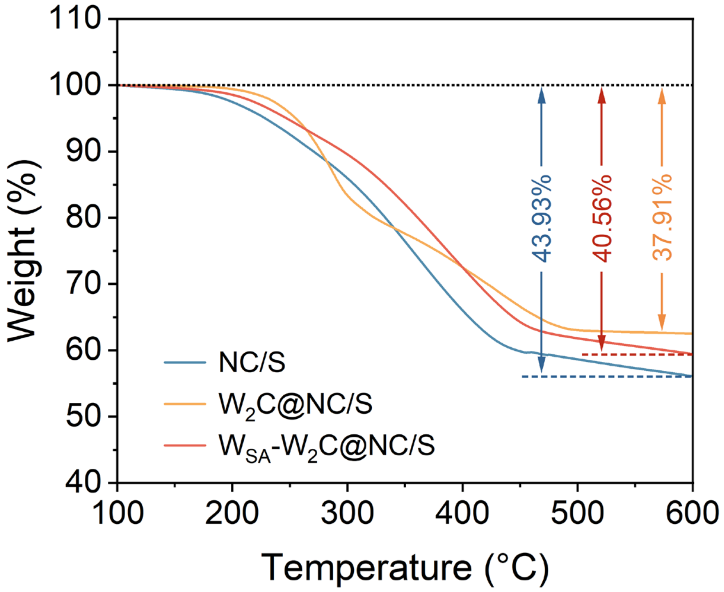

Supplementary Figure 19. Sulfur content analysis of the composites. Thermogravimetric analysis (TGA) curves of NC/S, W_2C@NC/S and W_{SA}-W_2C@NC/S tested under a nitrogen flow from 100 to 600 ℃ at a rate of 10 ℃ min^{-1}.

Supplementary Table 2. The analysis of sulfur contents in control samples in previous Na/K-S literatures.

Battery system	Control samples	Sulfur content	Difference	Measurement	Reference
K-S batteries	S@Co-NC<br>S@NC<br>S@SA-NC	49.3%<br>51.2%<br>56.8%	5.6%	TGA	*J. Am. Chem. Soc.* 143, 16902-16907 (2021)
	S@Cu-N_4<br>S@NC	56.7 %<br>50.7%	6%	TGA	*Angew. Chem. Int. Ed.* 62, e202301681 (2023)
Na-S batteries	S@HC<br>S@CoS_2/NC<br>S@Co_1-CoS_2/NC	34%<br>50%<br>54%	16%	TGA	*Angew. Chem. Int. Ed.* 61, e202200384 (2022)
	Mn_1@NC@S<br>Fe_1@NC@S<br>Ni_1@NC@S<br>Ge_1@NC@S<br>Pt_1@NC@S<br>Ru_1@NC@S	40%<br>68%<br>46%<br>17%<br>54%<br>53%	29%	TGA	*Angew. Chem. Int. Ed.* 132, 22355-22362 (2020)
	S@$FeNi_3$@HC<br>S@Ni@HC<br>S@HC	42%<br>42.5%<br>50.6%	8.1%	TGA	*ACS Nano* 15, 15218-15228 (2021)
	core−shell ZnS@S<br>ZCS@S<br>CoS_2@S	47%<br>57%<br>58%	10%	TGA	*ACS Nano* 14, 7259-7268 (2020)
	S/TiN-TiO_2@ MCCFs<br>S/MCCFs	56.9%<br>51.5%	5.4%	TGA	*ACS Nano* 15, 5639-5648 (2021)
	MCPS1<br>MCPS2	47%<br>63%	16%	TGA	*Nat. Commun.* 7, 11722 (2016)
	S/CoHC<br>S@Co_n-HC<br>S@HC	48%<br>47%<br>30%	17%	TGA	*Nat. Commun.* 9, 4082 (2018)
	NPCTs/S<br>NiS_2@NPCTs/S	47%<br>56%	9%	TGA	*Nat. Commun.* 10, 4793 (2019)
	S@NCFs<br>S@Ni-NCFs	45%<br>36%	9%	TGA	*Adv. Sci.* 7, 1902617 (2020)
	MMPCS-700@S<br>MMPCS-800@S<br>MMPCS-900@S	40.3%<br>43.8%<br>36.0%	3.7%	TGA	*Adv. Mater.* 34, 2108363 (2022)
	CN/Au/S<br>CN/S	56.5%<br>52.9%	3.6%	TGA	*Energy Environ. Sci.* 13, 562-570 (2020)
	Y SAs/NC-S<br>NC-S	67.4%<br>64.1%	3.3%	TGA	*J. Am. Chem. Soc.* 144, 18995-19007 (2022)
	155S<br>300S	72%<br>44%	28%	TGA	*Nano-Micro Lett.* 13, 121 (2021)

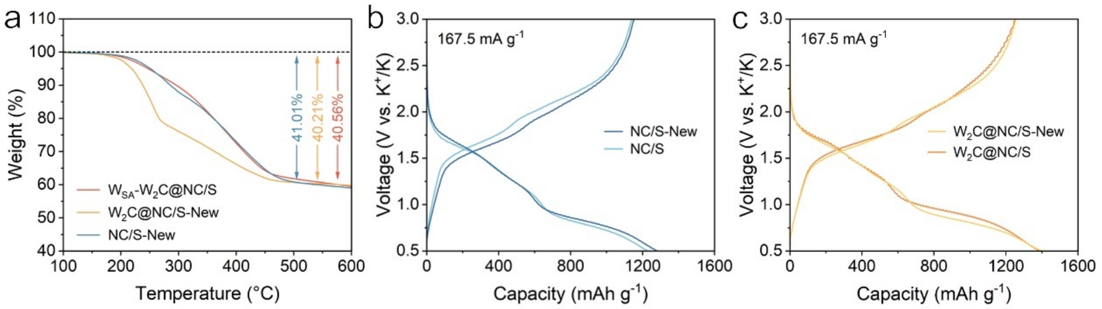

Supplementary Figure 20. Characterization and electrochemical performances of some sulfur cathodes. a TGA data of NC/S-New, W_2C@NC/S-New and W_{SA}-W_2C@NC/S. **b** GCD profiles of NC/S and NC/S-New. **c** GCD profiles of W_2C@NC/S and W_2C@NC/S-New.

Supplementary Table 3. Comparison between W_{SA}-W_2C@NC/S and the state-of-the-art cathodes for KSBs.

Cathodes	Sulfur content (%)	Sulfur loading (mg cm^{-2})	Maxmum Sulfur utilization	Rate performance (Capacity (mAh g^{-1}) @ Specific current (mA g^{-1}))	Ref.
W_{SA}-W_2C@NC/S	40.56%	~1.0/ ~2.8	89.8%/ 88.2%	1504 @ 167.5<br>1214 @ 837.5<br>1059 @ 1675	This work
C/S composite	18.6%	0.5−1.0	77.2%	1293.3 @ 20<br>741.2 @ 2000	10
PCNF/S	25%	0.5−1.0	83.0%	1392 @ 20<br>1138 @ 200	11
S@P-NCF	37%	~0.8	87.7%	1470 @ 337<br>560 @ 3370	12
SPAN	39.52%	*Not reported*	31.7%	532 @ 35	13
SPAN	38%	~1.0	42.4%	710 @ 47.5<br>218 @ 285	14
SPAN	36%	~0.8	58.9%	987 @ 837.5	15
I-S@pPAN	~42%	~1.0	86.1%	1442 @ 167.5	16
FS-SPAN	33%	~0.8	80.2%	1345 @ 70<br>680 @ 1000	17
γS-CNFs	~50%	0.69	52.8%	885 @ 167.5<br>66 @ 3350	18
CCS	39.25%	0.39–0.59	26.9%	440 @ 150<br>94 @ 1000	19
EAMC12	37.8%	1.5−2.0	69.5%	1165 @ 335<br>572 @ 1675	20

S@SA-NC	51.2%	~1.0	75.6%	1266 @ 337<br>868 @ 837.5	21
CCS@CBC_450<br>(3 M KTFSI in TEGDME)	40%	~1.5	42.4	711 @ 100<br>130 @ 1000	22
S/CNF<br>(1 M KCF_3SO_3 in TEGDME)	27.3%	~1.0	67.2%	1126 @ 167.5<br>938 @ 335<br>780 @ 558.3	23
CNT/S<br>(3 M KFSI in DME)	25.5%	~0.2	18.6%	311 @ 50<br>94 @ 500	24
ACF-1500@S<br>(3 M KFSI in DME)	15%	~1.3	18.1%	303 @ 50	25
S_8/VC<br>(0.3 M $Cu(TFSI)_2$-0.1 M KTFSI in Me-Im)	*Not reported*	1–1.2	50.6%	847 @ 150	26
0.05 M $K_2S_{5\sim6}$ catholyte<br>(0.5 M KTFSI in DEGDME)	*Not reported*	*Not reported*	22.3%	374 @ 55.8<br>107 @ 1116	27
0.02 M K_2S_5 catholyte<br>(1 M KTFSI in DEGDME)	*Not reported*	~0.128	33.2%	556 @ 117	28

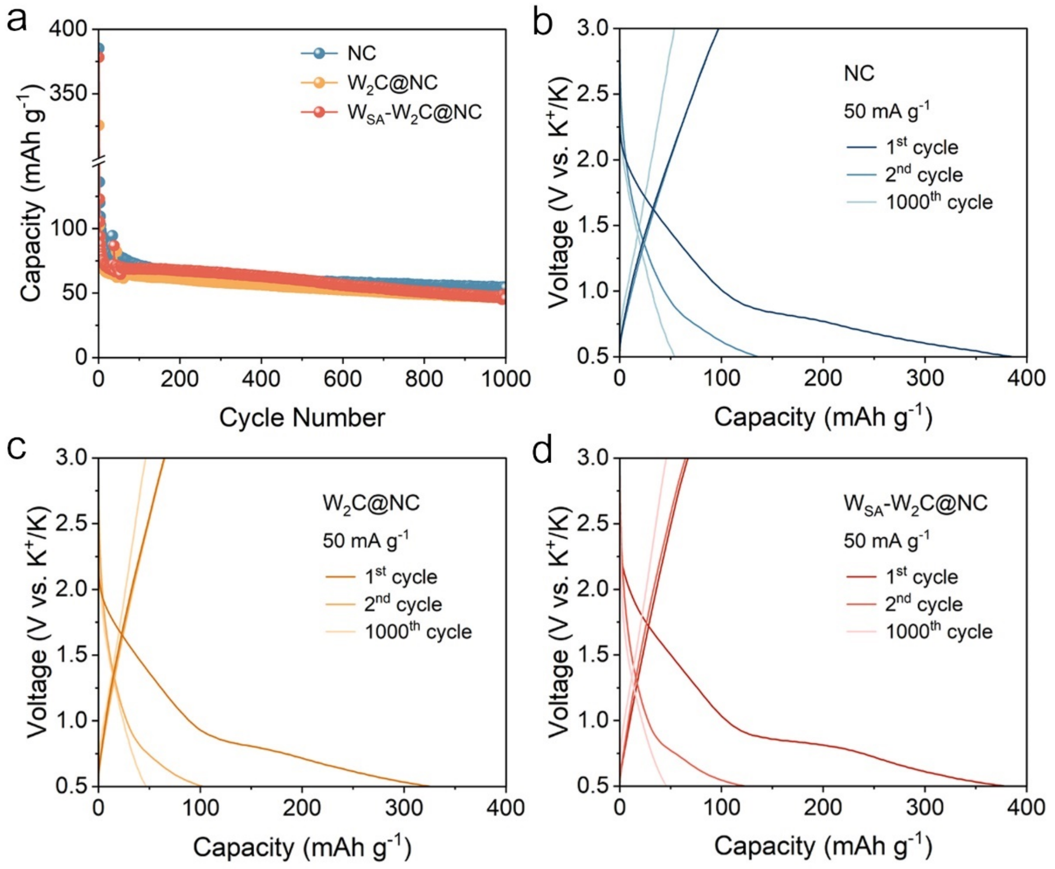

Supplementary Figure 21. Electrochemical performances of pure hosts. a Cyclability of NC, W_2C@NC and W_{SA}-W_2C@NC pure hosts at 50 mA g^{-1} for 1000 cycles. Galvanostatic charge-discharge profiles curves of the **b** NC, **c** W_2C@NC and **d** W_{SA}-W_2C@NC electrode at 50 mA g^{-1} for the first two cycles and 1000th cycle.

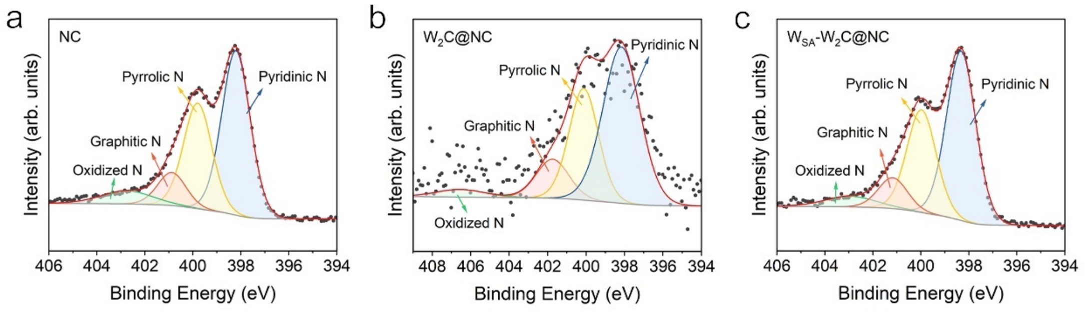

Supplementary Figure 22. **Nitrogen element analysis of the hosts.** High-resolution N 1s XPS spectra of **a** NC, **b** W_2C@NC and **c** W_{SA}-W_2C@NC.

Supplementary Table 4. Nitrogen composition in different hosts according to high-resolution N 1s XPS spectra.

	W_{SA}-W_2C@NC	W_2C@NC	NC
Pyrrolic N	52.6%	51.1%	50.3%
Pyridinic N	32.0%	32.0%	32.3%
Graphitic N	9.6%	12.1%	10.1%
Oxidized N	5.8%	4.8%	7.3%

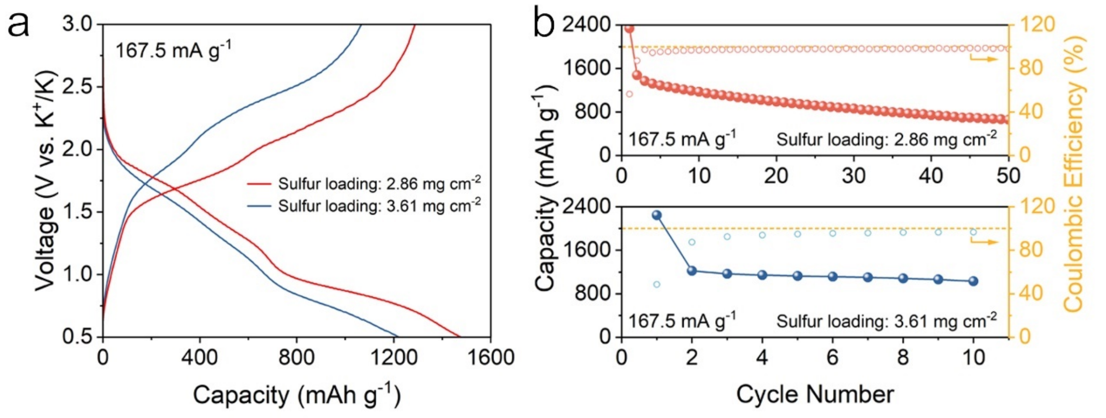

Supplementary Figure 23. Electrochemical performances of W_{SA}-W_2C@NC/S cathodes with high sulfur loading. a GCD profiles curves at 167.5 mA g^{-1} and **b** cyclability of KSBs employing W_{SA}-W_2C@NC/S cathodes with high sulfur loadings.

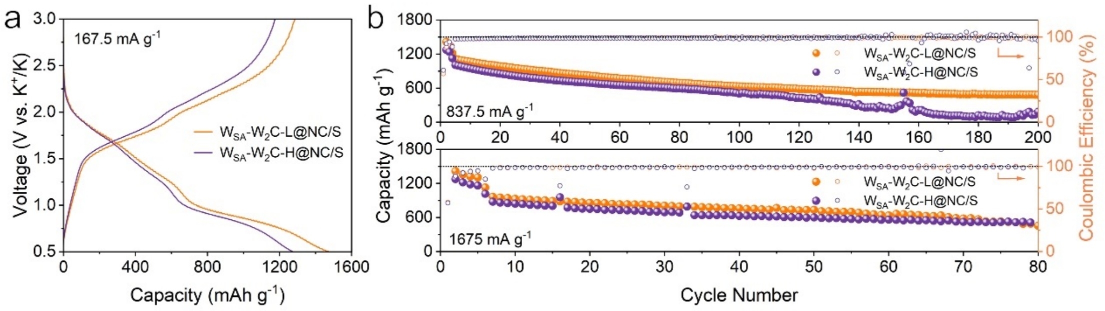

Supplementary Figure 24. Electrochemical performances of W_{SA}-W_2C-H@NC/S and W_{SA}-W_2C-L@NC/S cathodes. Electrochemical performance of KSBs employing W_{SA}-W_2C-L@NC/S and W_{SA}-W_2C-H@NC/S cathodes. **a** GCD curves at 167.5 mA g^{-1}. **b** Cyclability at 837.5 and 1675 mA g^{-1}.

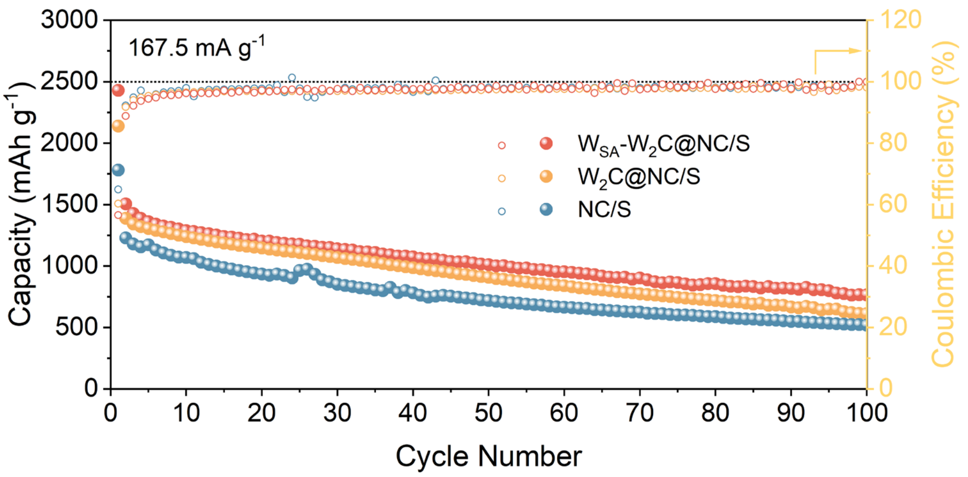

Supplementary Figure 25. Electrochemical performances of different cathodes at 167.5 mA g^{-1}. Cyclability at 167.5 mA g^{-1} of KSBs employing NC/S, W_2C@NC/S and W_{SA}-W_2C@NC/S cathodes.

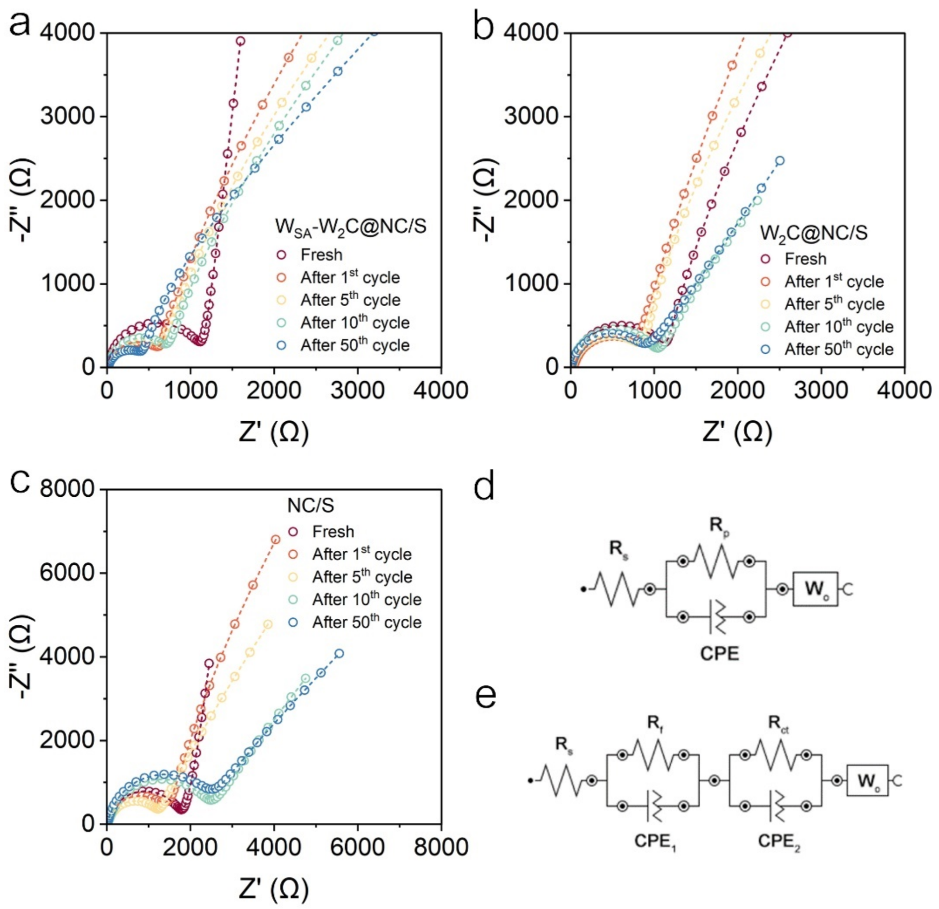

Supplementary Figure 26. Impedance analysis of KSBs employing different sulfur cathodes. EIS spectra of KSBs with **a** W_{SA}-W_2C@NC/S, **b** W_2C@NC/S, **c** NC/S cathodes after different cycles at 167.5 mA g^{-1} and **d,e** corresponding equivalent circuit diagrams.

Supplementary Table 5. EIS fitting results of KSBs with W_{SA}-W_2C@NC/S, W_2C@NC/S and NC/S cathodes after different cycles at 167.5 mA g^{-1}.

Sample	W_{SA}-W_2C@NC/S				W_2C@NC/S				NC/S			
	R_s	R_p	R_f	R_{ct}	R_s	R_p	R_f	R_{ct}	R_s	R_p	R_f	R_{ct}
Fresh	7.80	1106	*NA*	*NA*	9.57	1128	*NA*	*NA*	5.82	1722	*NA*	*NA*
After 1st cycle	6.01	*NA*	42.4	557	4.13	*NA*	41.9	770	5.65	*NA*	49.6	1319
After 5th cycle	5.24	*NA*	47.5	576	3.45	*NA*	140	841	5.62	*NA*	53.3	1155
After 10th cycle	4.86	*NA*	54.2	697	4.18	*NA*	272	1025	3.77	*NA*	72.4	2348
After 50th cycle	5.08	*NA*	13.4	427	4.50	*NA*	309	731	4.72	*NA*	66.8	2448

[a] R_s: Ohmic resistance. [b] R_p: Interface resistance for fresh cell. [c] R_f: Interface resistance by CEI. [d] R_{ct}: Charge transfer resistance.

Electrochemical impedance spectroscopy (EIS) was performed on KSBs with different cathodes over numerous cycling cycles to investigate the behavior of electrode durability in more depth. As shown in Supplementary Fig. 24 and Supplementary Table 3, before cycling, the KSBs employing the W_{SA}-W_2C@NC/S cathode exhibited the lowest resistance (R_p, 1106 Ω) and the largest slope in the low-frequency Warburg diffusion range compared to the W_2C@NC/S (1128 Ω) and NC/S (1722 Ω), indicating the fastest diffusion of potassium ions in W_{SA}-W_2C@NC/S cathode. After cycling at 167.5 mA g^{-1}, KSBs with W_{SA}-W_2C@NC/S cathode remained an extremely low CEI impedance (R_f) and charge transfer impedance (R_{ct}). The resistance kept increasing during the cycling for NC/S and W_2C@NC/S cells, which is in line with the faster decay in capacity upon cycling. Therefore, the key to the superior performance of W_{SA}-W_2C@NC/S over the control groups is the improved ionic diffusivity and electron conductivity of the host, which facilitates the surface diffusion and subsequent conversion of KPSs. In particular, the W_{SA}-W_2C@NC/S cathode maintained a low impedance (427 Ω) after 50 cycles with W_2C@NC/S and NC/S as high as 731 Ω and 2448 Ω, respectively, which means that hosts with W_{SA}-W_2C sites did not have a significant accumulation of inactivated solid-phase KPSs.

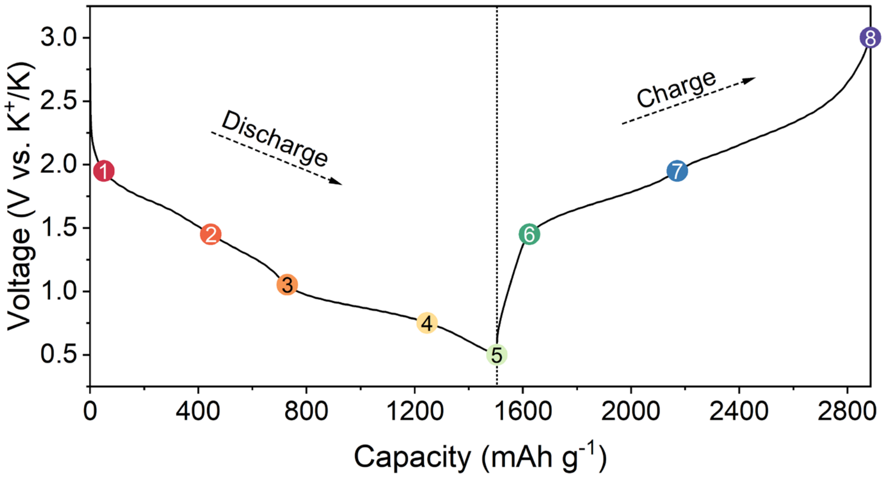

Supplementary Figure 27. GCD profiles of W_{SA}-W_2C@NC/S cathode. GCD profiles of the KSB with W_{SA}-W_2C@NC/S cathode at 167.5 mA g^{-1} for the second cycle. The potentials at which the *ex-situ* SAED patterns and *ex-situ* XPS spectra collected are marked by colored spheres.

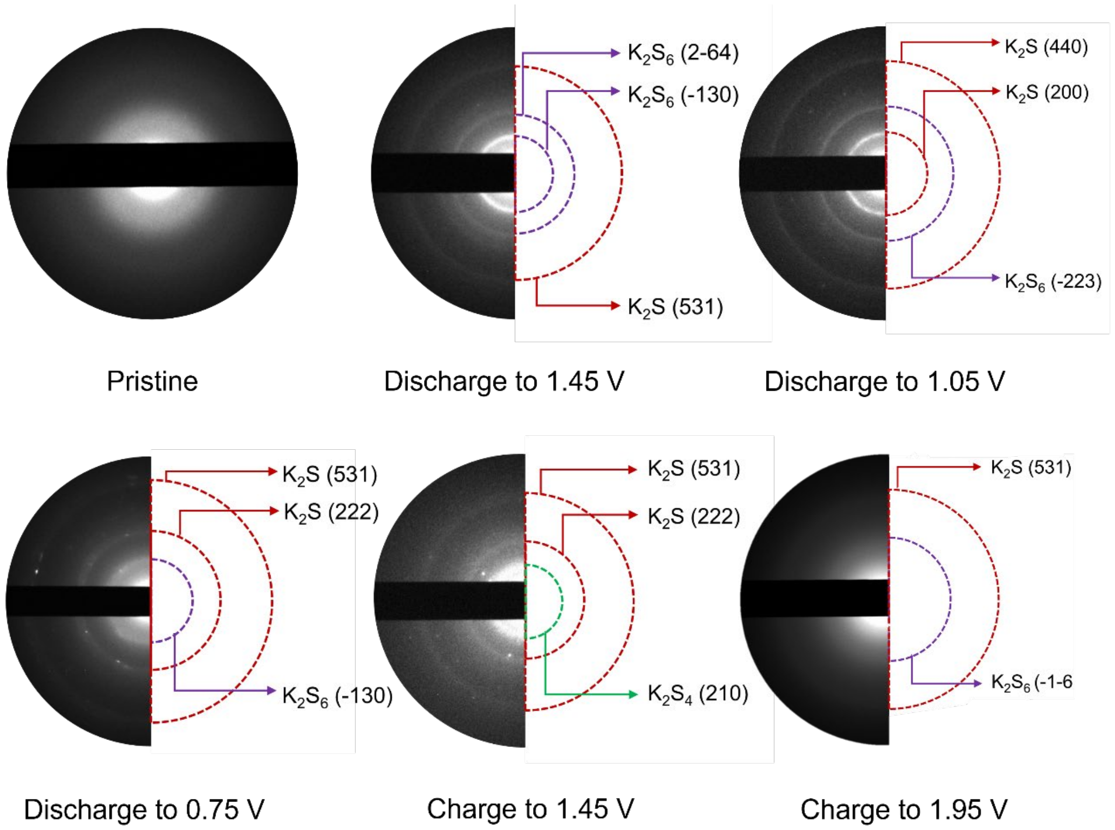

Supplementary Figure 28. *Ex-situ* SAED patterns for W_{SA}-W_2C@NC/S cathode at various voltages. *Ex-situ* SAED patterns for W_{SA}-W_2C@NC/S cathode at pristine state, discharge states of Dis-1.45 V, 1.05 V, 0.75 V, and charge states of Cha-1.45 V, 1.95 V.

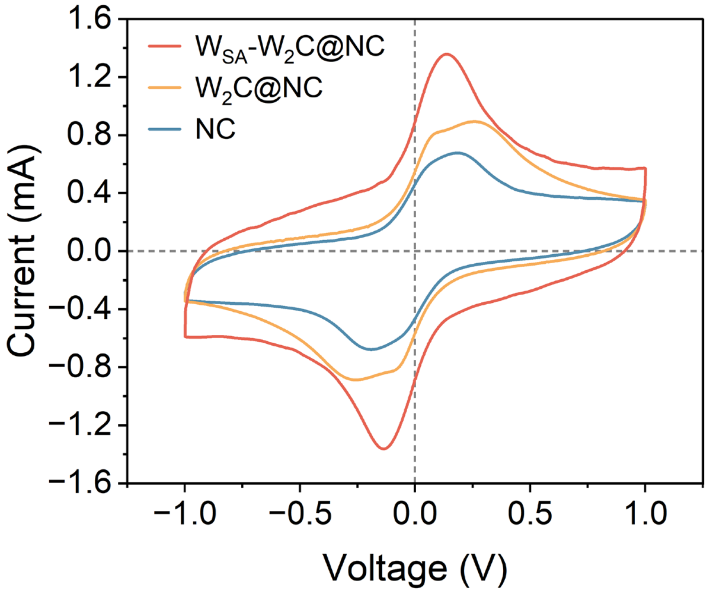

Supplementary Figure 29. CV curves for K_2S_6 symmetric cells. CV curves for at 25 mV s^{-1} K_2S_6 symmetric cells with NC, W_2C@NC, and W_{SA}-W_2C@NC electrodes.

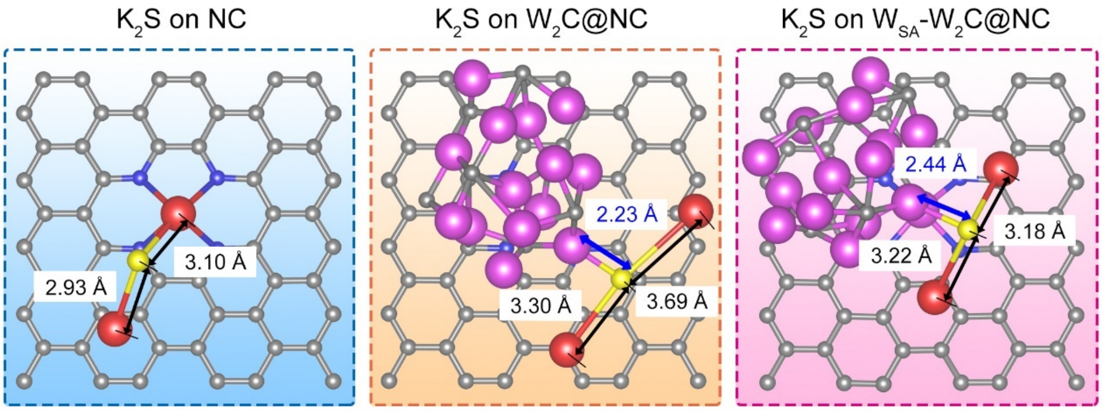

Supplementary Figure 30. Configurations during K_2S dissociation on the hosts. Transition state configurations of K_2S dissociation on W_{SA}-W_2C@NC, W_2C@NC and NC.

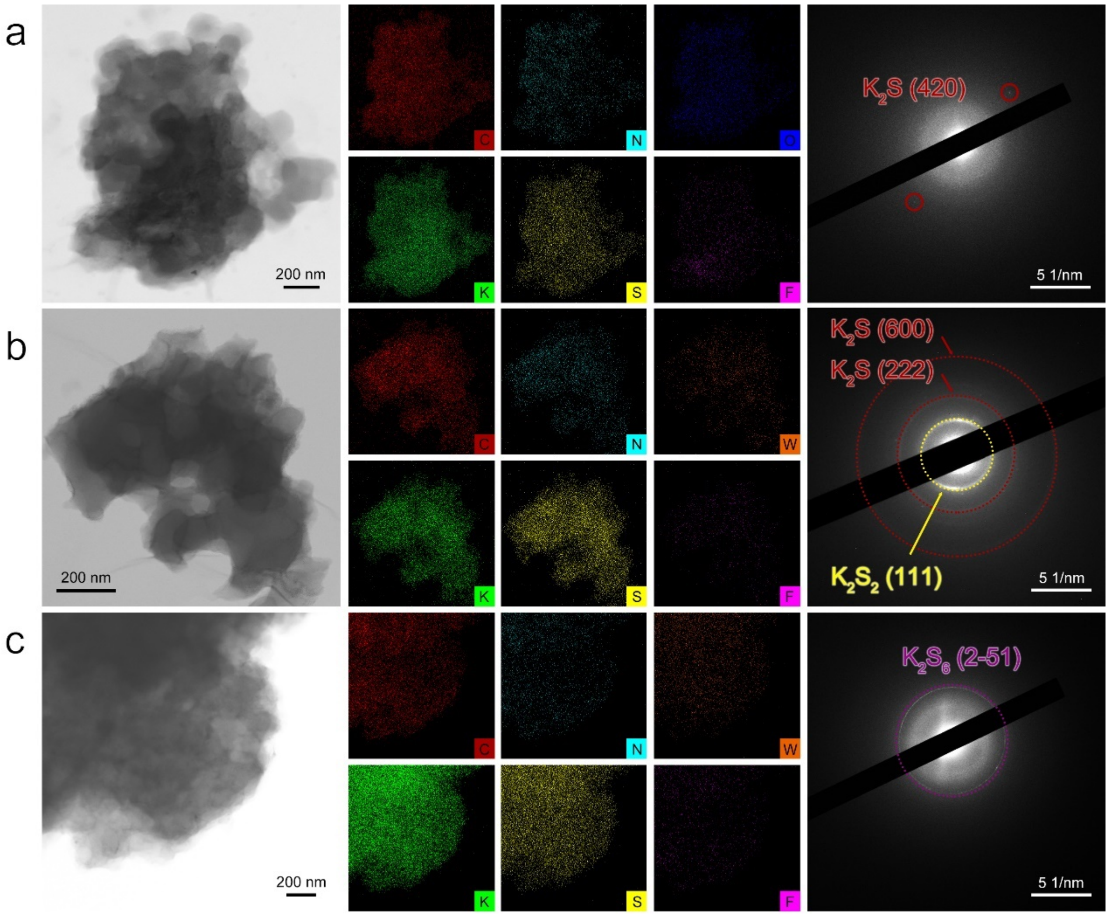

Supplementary Figure 31. Morphology, element distribution and phase information of cycled cathodes. TEM images and corresponding EDS elemental maps, SAED patterns of **a** NC/S cathode, **b** W_2C@NC/S cathode, and **c** W_{SA}-W_2C@NC/S cathode after 97 cycles at 1675 mA g^{-1}.

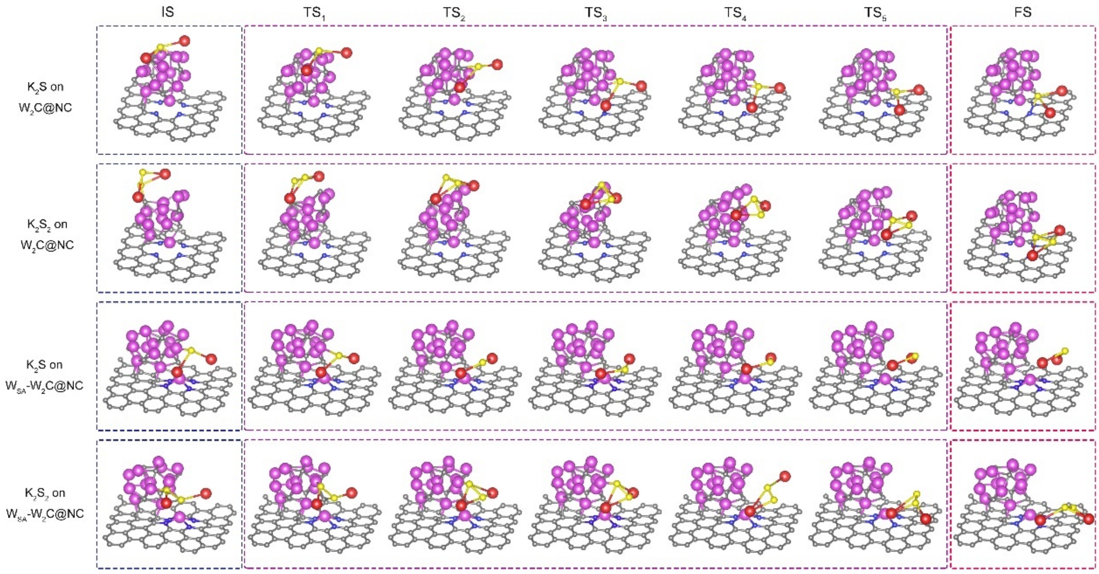

Supplementary Figure 32. Configuration evolution of solid-phase polysulfides migrating from the catalytic site to the substrate on the hosts. The initial, transition, final configurations of K_2S and K_2S_2 migration from W_2C catalytic site to the substrates for W_2C@NC and W_{SA}-W_2C@NC hosts.

Supplementary References

1. Perdew, J. P., Burke, K. & Ernzerhof, M. Generalized gradient approximation made simple. *Phys. Rev. Lett.* **77**, 3865 (1996).
2. Hammer, B., Hansen, L. B. & Nørskov, J. K. Improved adsorption energetics within density-functional theory using revised Perdew-Burke-Ernzerhof functionals. *Phys. Rev. B* **59**, 7413 (1999).
3. Blöchl, P. E. Projector augmented-wave method. *Phys. Rev. B* **50**, 17953 (1994).
4. Kresse, G. & Joubert, D. From ultrasoft pseudopotentials to the projector augmented-wave method. *Phys. Rev. B* **59**, 1758 (1999).
5. Monkhorst, H. J. & Pack, J. D. Special points for Brillouin-zone integrations. *Phys. Rev. B* **13**, 5188 (1976).
6. Henkelman, G. & Jónsson, H. Improved tangent estimate in the nudged elastic band method for finding minimum energy paths and saddle points. *J. Chem. Phys.* **113**, 9978 (2000).
7. Henkelman, G., Uberuaga, B. P. & Jónsson, H. A climbing image nudged elastic band method for finding saddle points and minimum energy paths. *J. Chem. Phys.* **113**, 9901 (2000).
8. Sheppard, D. & Henkelman, G. Paths to which the nudged elastic band converges. *J. Comput. Chem.* **32**, 1769 (2011).
9. Hansen, U., Vogl, P. & Fiorentini, V. Quasiharmonic versus exact surface free energies of Al: A systematic study employing a classical interatomic potential. *Phys. Rev. B* **60**, 5055 (1999).
10. Xiong, P. et al. Room-temperature potassium–sulfur batteries enabled by microporous carbon stabilized small-molecule sulfur cathodes. *ACS Nano* **13**, 2536 (2019).
11. Zhao, X. et al. High performance potassium–sulfur batteries and their reaction mechanism. *J. Mater. Chem. A* **8**, 10875 (2020).
12. Liang, J., Song, W., Wang, H., Ding, J. & Hu, W. Porosity engineering towards nitrogen-rich carbon host enables ultrahigh capacity sulfur cathode for room temperature potassium-sulfur batteries. *Nanomaterials* **12**, 3968 (2022).
13. Zhang, Y. et al. A novel rechargeable potassium–sulfur battery based on liquid alloy anode. *Mater. Lett.* **242**, 5 (2019).
14. Liu, Y. et al. Sulfur nanocomposite as a positive electrode material for rechargeable potassium–sulfur batteries. *Chem. Commun.* **54**, 2288 (2018).
15. Hwang, J.-Y., Kim, H. M. & Sun, Y.-K. High performance potassium–sulfur batteries based on a sulfurized polyacrylonitrile cathode and polyacrylic acid binder. *J. Mater. Chem. A* **6**, 14587 (2018).
16. Ma, S. et al. Iodine-doped sulfurized polyacrylonitrile with enhanced electrochemical performance for room-temperature sodium/potassium sulfur batteries. *Chem. Commun.* **55**, 5267 (2019).
17. Yang, K., Kim, S., Yang, X., Cho, M. & Lee, Y. Binder-free and high-loading cathode realized by hierarchical structure for potassium–sulfur batteries. *Small Methods* **6**, 2100899 (2022).
18. Pai, R., Cardoza, N. A., Natu, V., Barsoum, M. W. & Kalra, V. A non-confined gamma

monoclinic sulfur cathode in carbonate electrolyte based room temperature K–S batteries. *J. Mater. Chem. A* **11**, 15924 (2023).

19. Ma, R., Fan, L., Wang, J. & Lu, B. Confined and covalent sulfur for stable room temperature potassium-sulfur battery. *Electrochim. Acta* **293**, 191 (2019).
20. Hu, L. et al. A superficial sulfur interfacial control strategy for the fabrication of a sulfur/carbon composite for potassium–sulfur batteries. *Chem. Commun.* **57**, 1490 (2021).
21. Ye, C. et al. Catalytic oxidation of K_2S via atomic Co and pyridinic N synergy in potassium–sulfur batteries. *J. Am. Chem. Soc.* **143**, 16902 (2021).
22. Bharti, V. K., Pathak, A. D., Anjan, A., Sharma, C. S. & Khandelwal, M. Covalently confined sulfur composite with carbonized bacterial cellulose as an efficient cathode matrix for high-performance potassium–sulfur batteries. *ACS Sustain. Chem. Eng.* **10**, 16634 (2022).
23. Yu, X. & Manthiram, A. A reversible nonaqueous room-temperature potassium-sulfur chemistry for electrochemical energy storage. *Energy Stor. Mater.* **15**, 368 (2018).
24. Yuan, X. et al. High-Performance Stable Potassium–Sulfur Batteries Enabled by Free-Standing CNT Film-Based Composite Cathodes. *J. Electron. Mater.* **50**, 3037 (2021).
25. Yuan, X. et al. Free-standing, flexible and stable potassium–sulfur battery enabled by controllable porous carbon cloth. *J. Power Sources* **480**, 228874 (2020).
26. Lai, N.-C., Cong, G. & Lu, Y.-C. A high-energy potassium–sulfur battery enabled by facile and effective imidazole-solvated copper catalysts. *J. Mater. Chem. A* **7**, 20584 (2019).
27. Hwang, J.-Y., Kim, H. M., Yoon, C. S. & Sun, Y.-K. Toward high-safety potassium–sulfur batteries using a potassium polysulfide catholyte and metal-free anode. *ACS Energy Lett.* **3**, 540 (2018).
28. Shao, J. et al. K3SbS4 as a Potassium Superionic Conductor with Low Activation Energy for K–S Batteries. *Angew. Chem. Int. Ed.* **61**, e202200606 (2022).
